# Mitotic HOOK3 phosphorylation by ERK1c drives microtubule-dependent Golgi destabilization and fragmentation

**DOI:** 10.1016/j.isci.2021.102670

**Published:** 2021-05-31

**Authors:** Inbal Wortzel, Galia Maik-Rachline, Suresh Singh Yadav, Tamar Hanoch, Rony Seger

**Affiliations:** 1 Department of Biological Regulation, Weizmann Institute of Science, Rehovot 7610001, Israel

**Keywords:** cell biology, functional aspects of cell biology

## Abstract

ERK1c is an alternatively spliced isoform of ERK1 that specifically regulates mitotic Golgi fragmentation, which allows division of the Golgi during mitosis. We have previously shown that ERK1c translocates to the Golgi during mitosis where it is activated by a resident MEK1b to induce Golgi fragmentation. However, the mechanism of ERK1c functions in the Golgi remained obscure. Here, we searched for ERK1c substrates and identified HOOK3 as a mediator of ERK1c-induced mitotic Golgi fragmentation, which requires a second phosphorylation by AuroraA for its function. In cycling cells, HOOK3 interacts with microtubules (MTs) and links them to the Golgi. Early in mitosis, HOOK3 is phosphorylated by ERK1c and later by AuroraA, resulting in HOOK3 detachment from the MTs, and elevated interaction with GM130. This detachment modulates Golgi stability and allows fragmentation of the Golgi. This study demonstrates a novel mechanism of Golgi apparatus destabilization early in mitosis to allow mitotic progression.

## Highlights

• HOOK3 is a Golgi fragmentation-related substrate of ERK1c
• ERK1c phosphorylates HOOK3 on Ser238 and then AuroraA phosphorylates Ser707
• Doubly phosphorylated HOOK3 detaches from microtubules and interacts with GM130
• These changes destabilize the Golgi during mitosis and induce its fragmentation

## Graphical abstract

**Figure:**
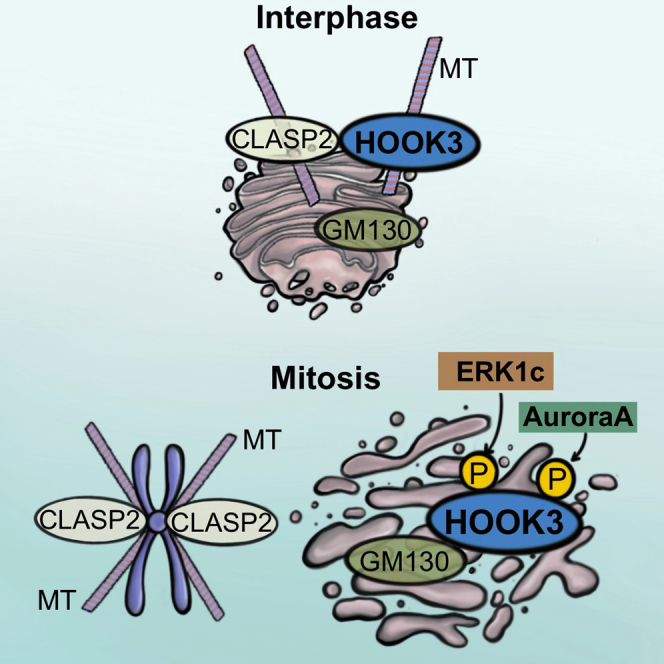

## Introduction

Mitotic Golgi fragmentation is a central process in mammalian cells that allows division of the Golgi apparatus into the newly formed daughter cells, and allows proper mitotic progression (Ayala and Colanzi, 2017; Champion et al., 2017; Shorter and Warren, 2002). In interphase, the Golgi apparatus is organized into stacks of cisternae that are interconnected to form a ribbon-like structure, localized as a condensed structure adjacent to the nuclei (Makhoul et al., 2018). This structure is well known for its function in protein trafficking and post-translational modification of membranal or secreted proteins (McCaughey and Stephens, 2019). During this time, the intact Golgi structure is stabilized by several mechanisms: (i) structural proteins such as Golgins, Golgi reassembly stacking proteins (GRASPs), and GM130 that interact with each other to maintain the structure, (ii) cytoskeletal elements such as microtubules (MTs) that determine the localization of the Golgi at the perinuclear region (Kulkarni-Gosavi et al., 2019; Rios and Bornens, 2003), and prevent its disintegration (Thyberg and Moskalewski, 1999), (iii) regulatory kinases that phosphorylate these and other Golgi components to dictate Golgi integrity (Chia et al., 2012), (iv) membranes that surround the organelle whose integrity is ensured by a constant phospholipid supply from the ER (Marra et al., 2007). However, in mitosis, the Golgi apparatus does not participate in trafficking anymore and undergoes a rapid fragmentation (Cabrera-Poch et al., 1998). The process of this fragmentation is composed of two main steps. The first is the Golgi unlinking, which occurs at the end of G2 and in prophase (Ayala and Colanzi, 2017; Ayala et al., 2020). In this stage, the Golgi is separated into several isolated stacks that are usually distributed around the nucleus. The second step is the dispersal of the stacks into thousands of small vesicles localized all over the mitotic cell, which occurs during prometaphase and lasts through anaphase. Some of these vesicles may fuse to the ER or other membranes, but many of the vesicles remain dispersed within the cell (Ayala et al., 2020; Champion et al., 2017). Toward the end of mitosis (telophase), the small vesicles merge back together to form a full-size Golgi apparatus in each of the newly formed daughter cell.

Being such a central process that promotes proper cell division (Corda et al., 2012; Hidalgo Carcedo et al., 2004; Preisinger et al., 2005; Sutterlin et al., 2002), the mitotic Golgi fragmentation is tightly regulated by multiple factors. Thus, the first stage of the fragmentation involves mainly the phosphorylation of Golgi structural proteins such as GRASP55 and GRASP65, which is mainly mediated by signaling protein kinases such as ERKs, JNKs, or other kinases downstream of MEK (Acharya et al., 1998; Cervigni et al., 2015; Cha and Shapiro, 2001; Jesch et al., 2001; Yoshimura et al., 2005). At the same time, the connecting membranous extensions are cleaved to allow the separation of the stacks. This process may involve the membrane fission protein BARS (Ayala et al., 2020; Hidalgo Carcedo et al., 2004) that is recruited to the Golgi by 14-3-3γ/PI4K (Valente et al., 2012). In the second step, namely the vesiculation, the structural proteins GRASP55/65 are further phosphorylated by CDK1 (Tang et al., 2012), as well as other protein kinases such as PLK1, PLK3 and MYT1 (Lopez-Sanchez et al., 2009; Sengupta and Linstedt, 2010; Villeneuve et al., 2013). CDK1 also phosphorylates Golgins and other Golgi proteins to allow their detachment from structural Golgi protein or membrane complexes to destabilize the Golgi stacks (Zhang and Wang, 2015). Such destabilizing detachment of Golgi proteins was shown also for Guanine-nucleotide exchange factor GBP that is phosphorylated by AMPK during mitosis, to induce Arf1 inactivation and thereby Golgi vesiculation (Altan-Bonnet et al., 2003; Lee et al., 2015). Throughout the two stages of fragmentation, the stabilizing association of the Golgi with MTs and other cytoskeletal elements is disrupted by several mechanisms that are not yet fully understood (Mironov and Beznoussenko, 2011). It is very likely that additional phosphorylations, such as the one executed by ERK1c are involved in destabilization of the Golgi apparatus, and thereby lead to its fragmentation. Finally, it was shown that the fragmentation may initiate mitotic-related signaling by activating protein kinases such as AuroraA (Persico et al., 2010) and VRK1 (Lopez-Sanchez et al., 2009) that further regulate mitotic entry and thereby are involved in the Golgi-induced mitotic regulation.

A group of proteins that were shown to be involved in the regulation of Golgi fragmentation are the components of the ERK cascade. The ERK cascade is a central signaling pathway that regulates various cellular processes including proliferation, differentiation, apoptosis, and mitosis (Maik-Rachline et al., 2019; Wortzel and Seger, 2011). It is composed of three tiers of protein kinases (MAP3K, MAPKK, and MAPK) that sequentially phosphorylate and activate each other in a linear manner. This induces activation of the ERKs, which exert the activity of the cascade. Being involved in these different, and even opposing processes, the specificity of the cascade is tightly regulated by various mechanisms (Murphy and Blenis, 2006; Shaul and Seger, 2007). One of these mechanisms is the existence of different isoforms and even distinct protein kinases in each tier of the cascade. One isoform at the MAPK level that extends the specificity of the cascade is ERK1c, which we identified as an alternatively spliced isoform of ERK1 (Aebersold et al., 2004; Shaul and Seger, 2006). Interestingly, we found that this protein kinase is involved in the regulation of mitotic Golgi fragmentation (Shaul and Seger, 2006), a processes that had previously been shown to act downstream of MEK1 but not its well-known substrates, ERK1 and ERK2, that are not found in this organelle (Acharya et al., 1998). Later, we showed that the ERK1c translocates to the Golgi during prophase by a mechanism that involves the phosphorylation of ERK1c by CDK1, followed by interaction with a PI4KIIIβ/14-3-3γ shuttling complex (Wortzel et al., 2015). In the Golgi, ERK1c is activated by MEK1b, an alternatively spliced isoform of MEK1 (Shaul et al., 2009), forming an alternative ERK cascade. This cascade specifically regulates mitotic Golgi fragmentation, although the mechanism of action of this regulation is still obscure.

In this study, we identified HOOK3, a ubiquitously expressed Golgi-localized protein, as an ERK1c target in the Golgi. This protein is known to interact with Golgi membranes and MTs to regulate Golgi localization and stability (Walenta et al., 2001). We found that this protein is phosphorylated not only by ERK1c but also by AuroraA during mitosis. We showed that during interphase, HOOK3 is associated with both the Golgi apparatus and MTs in its unphosphorylated form, but at the onset of mitosis, it is phosphorylated on Ser238 by ERK1c, which allows a subsequent phosphorylation on Ser707 by AuroraA. As a consequence, the phosphorylations induce detachment of HOOK3 from the MTs and facilitate its interaction with GM130. These changes enable the release of the Golgi membranes from the stabilizing MTs, thus contributing to destabilization of the Golgi structure during mitosis. Our results demonstrate a novel function of ERK1c and uncover its effect on the destabilization of the Golgi apparatus, which occurs by its dissociation from MTs that further drives the progression of Golgi fragmentation.

## Results

### Screening for Golgi-localized ERK1c substrates

**Figure 1:**
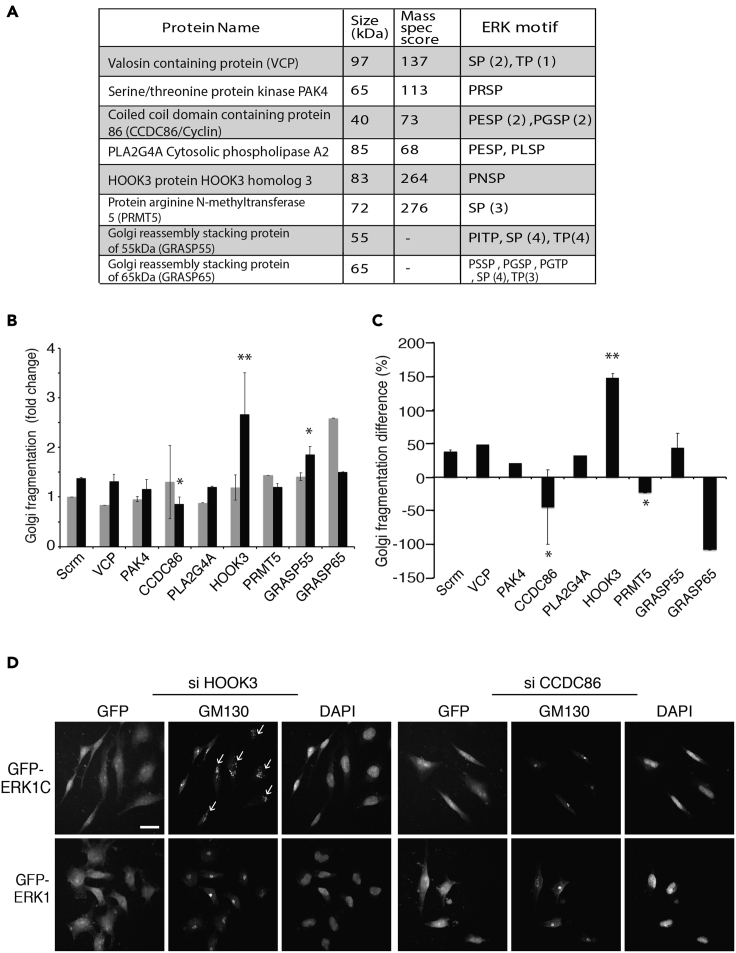
HOOK3 is a novel Golgi localized protein inducing Golgi fragmentation (A) A table summarizing top candidate ERK1c substrate proteins identified by mass spectrometry from sucrose gradient-enriched Golgi compartment from HeLa cells. The table contains the protein name, molecular weight, a mass spectrometry score (accuracy of peptide's mass compared to the calculated one. According to our experience with a large number of samples score above 60 is considered significant), and the ERK consensus phosphorylation motif. (B) HeLa cells were transfected with GFP-ERK1 or GFP-ERK1c for 24 hr, and then with 50 nM SiRNA against the indicated proteins for additional 48 hr. Cells were then fixed and stained with GM130 antibody. Golgi fragmentation rate from ERK1 (light gray) or ERK1C (black) transfected cells was calculated as a percent of cells with fragmented Golgi, out of the overexpressed cells (∗∗p < 0.01, ∗p < 0.05). (C) The percentage difference in Golgi fragmentation between ERK1 and ERK1c in each treatment. (D) Representative images from HOOK3 and CCDC86 SiRNA on HeLa cells, which were transfected with GFP-ERK1c or GFP-ERK1. Arrows indicate fragmented Golgi. Scale bar, 10μm. The experiments in B, C, and D were reproduced 3 times.

Golgi fragmentation during mitosis is not only important in dividing this organelle between two daughter cells but also serves as a mitotic driver (Corda et al., 2012). As such, this process is tightly regulated by a large number of protein kinases (Acharya et al., 1998; Cervigni et al., 2015; Cha and Shapiro, 2001), including ERK1c that migrates into the Golgi during mitosis to regulate the Golgi break-down (Shaul and Seger, 2006). Although it is clear that ERK1c plays a role in the process, the molecular mechanism by which active ERK1c exerts its function has yet to be defined. To investigate it, we first undertook to identify putative ERK1c substrates within the Golgi. For this purpose, we enriched the Golgi compartment from HeLa cells on sucrose gradient and used mass spectrometry to identify proteins that reside in this fraction. We identified more than 250 proteins, of which we concentrated on (i) the ones containing a full ERK consensus phosphorylation motif (Pro-Xaa-Ser/Thr-Pro) or three or more minimal consensus sites (Ser/Thr-Pro), (ii) high mass spectroscopy score (accuracy of peptides identified (>60)), and (iii) a known Golgi localization. Although it is likely that due to the stringent selection methods we missed some putative ERK1c substrates, the proteins that we did identify were chosen as leading compounds for further experiments (Figure 1A)

To find out which of the identified proteins is indeed a direct mediator or regulator of ERK1c outcomes, we first established a system in which we followed ERK1c effects on Golgi architecture. For this purpose, we used the ability of overexpressed ERK1c to induce Golgi fragmentation, independent of the cell cycle stages (Shaul and Seger, 2006). This use of non-mitotic Golgi fragmentation was important in order to follow the substrates that act specifically downstream of ERK1c, so the effect of other mitotic factors in this process would not mask it. Thus, HeLa cells were transfected with GFP-ERK1c, or GFP-ERK1 as a negative control, making sure that the level of overexpression of the two isoforms was equal. Then, Golgi fragmentation was measured by immunofluorescence staining of the Golgi marker GM130. The non-fragmented Golgi appears as one concentrated GM130 spot (∼2-3 μm) adjacent to the nucleus. Fragmented Golgi appears as a spread (>4 μm was considered as fragmented), less intense GM130 staining (arrows in Figures 1D and S1A), and in some cells it appeared broken to several smaller spots (Shaul and Seger, 2006; Wortzel et al., 2015). As expected, a significant portion of the ERK1c transfected cells had a fragmented Golgi (28%), while less Golgi fragmentation was seen also in non-transfected (∼5%, not shown), as well as in ERK1-transfected cells (17%). Thus, although overexpressed ERK1 can induce some fragmentation, probably due to its mis-localization, the homogeneous size of the non-transfected cells and the clear increase in size of the fragmented Golgi in the transfected cells allow the detection of ERK1c-specific processes.

Next, we knocked-down each of the identified proteins, as well as GRASP55 and GRASP65, which are well-known substrates of ERK1/2 (Acharya et al., 1998; Ayala and Colanzi, 2017; Cha and Shapiro, 2001; Jesch et al., 2001). Thus, HeLa cells overexpressing either ERK1 or ERK1c were treated with SiRNAs for the relevant proteins examined and indeed exhibited the expected decrease in protein or mRNA expression (Figures S1B and S1C). The knockdown was then followed by immunofluorescence quantification of Golgi fragmentation that revealed that while scramble SiRNA did not change the rate of fragmentation, the knockdowns of several tested proteins did affect it (Figures 1B–1D and S2). Thus, knockdown of CCDC86 slightly elevated the fragmentation in ERK1 overexpressing cells but significantly reduced the fragmentation in ERK1c-overexpressing ones. Knockdown of PRMT5 had minor effects on both ERK1/1c overexpressing cells, and the knockdown of HOOK3 profoundly increased fragmentation in ERK1c cells with only a minor effect on the ERK1 cells. Knockdown of GRASP55 elevated the fragmentation in both ERK1 and ERK1c cells, and that of GRASP65 elevated the fragmentation in ERK1, but had no effect on the ERK1c cells. Since overexpression of ERK1 induced elevation in Golgi fragmentation in some of the knockdowns, we also analyzed the results by presenting the difference in fragmentation between the ERK1c and ERK1-overexpressing cells (Figure 1C). These presentations made it clear that SiRNAs of CCDC86 and PRMT5 slightly reduced the fragmentation in ERK1c as compared to ERK1 overexpressing cells, while the most significant effects were observed with the knockdown of HOOK3 (Figures 1C and 1D). This knockdown of HOOK3 significantly increased the ERK1c-induced fragmentation, suggesting that it may have a negative role in the regulation of Golgi fragmentation. A rescue experiment with GFP-HOOK3 confirmed that the change in fragmentation is indeed due to the HOOK3 knockdown and not off-target effects (Figure S3). Interestingly, also GRASP65 knockdown reduced the ERK1c-induced fragmentation as compared to the ERK1-dependant one, but in this case, the effect was most likely due to elevation of fragmentation in ERK1 expressing cells, and not the actual reduction by ERK1c. Since the most significant effect on ERK1c-induced fragmentation was observed with Si-HOOK3, we decided to study the role of this protein in Golgi structure and morphology.

### HOOK3 depletion induces Golgi fragmentation

**Figure 2:**
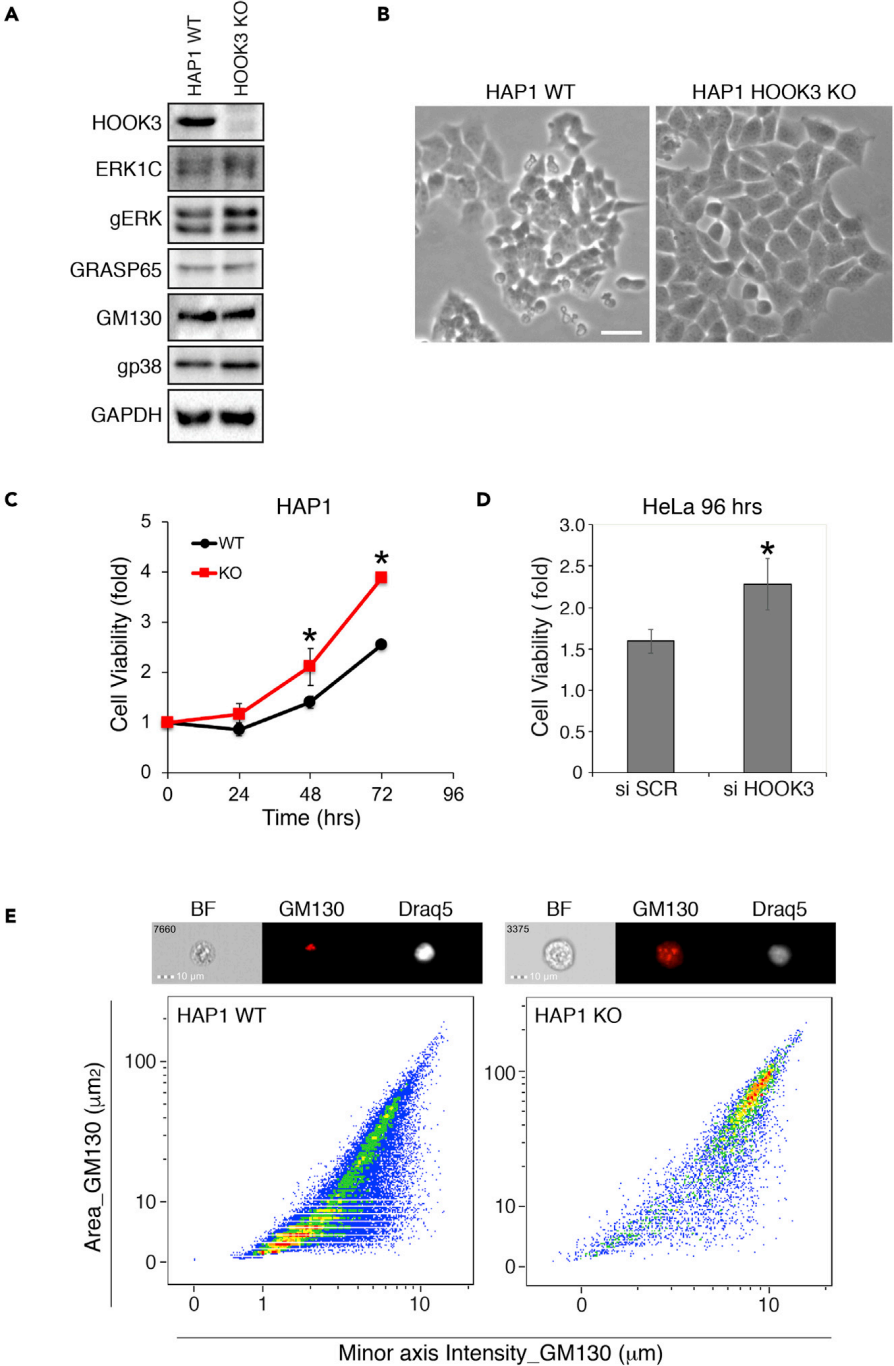
Characterization of HOOK3-deficient cells (A) HAP1 control (WT) and HOOK3 gene Crisper/Cas knockout cells (KO) were grown according to the manufacture instructions. Total cell lysates from both cell types (HAP1 WT and HOOK3 KO) were blotted against the indicated proteins and confirmed deletion of HOOK3 protein. (B) Representative images of grown cells morphology. Scale bar, 30μm. (C) Cell proliferation of control HAP1 (WT) and HOOK3 deleted (KO) cells following 72 hr maintenance in tissue culture is indicted. (D) Proliferation of HeLa cells initially transfected with scramble (SCR) or HOOK3 SiRNA followed by 96 hr maintenance in tissue culture. (E) The overall morphology of the Golgi apparatus was determined by imaging flow cytometry analysis method to quantify the rate of Golgi fragmentation in these cells. All experiments were reproduced 3 times (∗p < 0.05).

To investigate the role of HOOK3 in Golgi structure and cellular functions, we first used CRISPR/Cas9 to knockout HOOK3 in HAP1 cells in order to completely deplete HOOK3 without affecting the expression of other proteins (Figure 2A). Interestingly, the HOOK3-deficient cells presented a different cellular morphology, as they seemed bigger, and more spread out as compared to HOOK3 containing cells (Figure 2B). Further investigation revealed that the knockout cells were viable over multiple passages (data not shown), and their proliferation rate was significantly faster than the control HOOK3-WT cells (Figure 2C). Reducing HOOK3 expression by SiRNA in HeLa cells yielded a similar increase in cellular proliferation (Figure 2D). Finally, we examined the overall architecture of the Golgi using our previously developed flow cytometry analysis to quantify the rate of Golgi fragmentation (Wortzel et al., 2017). In this method, an intact Golgi detected by GM130 staining occupies a small area (Area_GM130), and its diameter is short (Minor axis intensity_GM130). Therefore, a cell with an intact Golgi is found in the area where both axis values are low, but fragmented Golgi will demonstrate a high value for both area and diameter. We found that in accordance with the results obtained with the SiRNA above, the Golgi morphology is generally intact in HAP1 control cells but mostly fragmented in the knockout cells (Figure 2E). Taken together, we concluded that HOOK3 is essential for the proper organization of the Golgi, and therefore, affects the Golgi fragmentation and its consequent mitotic progression. As a consequence, the rate of proliferation is increased in both the knock-out and the knockdown cells. However, the effect of the HOOK3 knockout on cell morphology likely indicates that HOOK3 may have other effects besides its role in regulating Golgi fragmentation. Hence, HOOK3 is likely to be a negative regulator of Golgi fragmentation, and thereby also proliferation. In order to avoid the possibility that the ERK1c and HOOK3 are not acting in the same signaling axis, we next tested if there is a kinase-substrate interaction between them.

### The role of ERK1c in a mitotic phosphorylation of HOOK3

**Figure 3:**
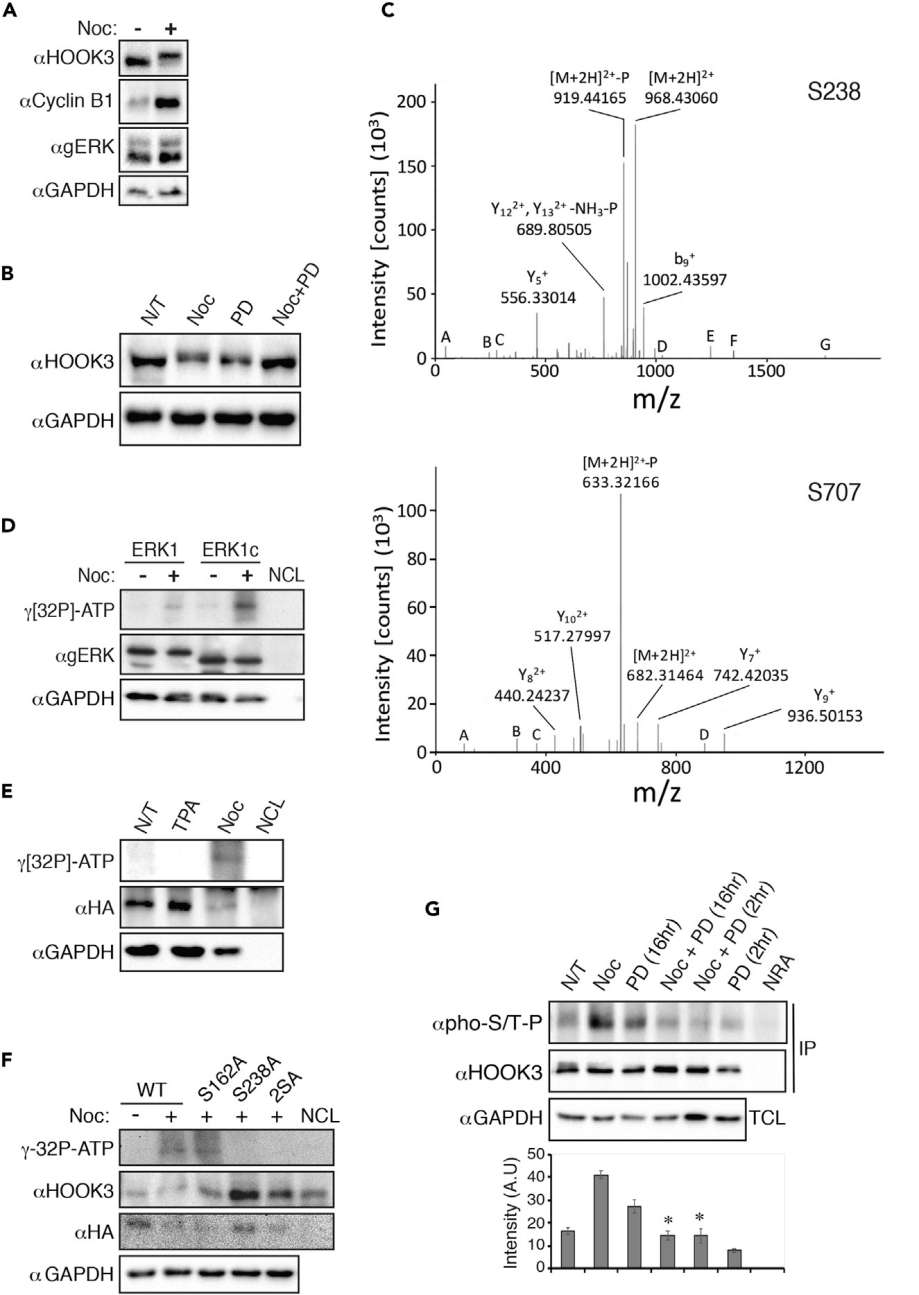
HOOK3 is phosphorylated by ERK1c during mitosis (A) Representative blots of total cells lysates from HeLa cells, which were treated with 100 ng/mL nocodazole for 16 hr (+) or left non-treated (−). In this case, the SDS-PAGE duration was longer (until the 50 kDa marker run out) in order to visualize the upshift of HOOK3. Noc – nocodazole. (B) Representative blots of total cells lysates from HeLa cells, which were treated with nocodazole in the presence of a MEK inhibitor. HeLa cells were either left non-treated (N/T) or treated with nocodazole (Noc, 100 ng/mL, 16 hr) with or without MEK inhibitor (PD, PD184352, 2μM for 2 hr). The SDS-PAGE was treated as described in 3A. (C) Cells were treated as described in panel A and were subjected to 10% SDS-PAGE followed by staining with InstantBlue solution. Upshifted nocodazole treated HOOK3 band was further analyzed by mass spectrometry and the main phospho-peptides detected are presented. (D) Representative autoradiogram (upper) and blot (lower) of ERK1 or ERK1c *in vitro* kinase assay using HOOK3 as a substrate. HeLa cells were transfected with HA-ERK1 or HA-ERK1c and were treated with 100 ng/mL nocodazole for 16 hr (+) or left non-treated (−). The cells were lysed, and the extracts were subjected to IP using anti HA antibody. The IPed kinase was then subjected to an *in vitro* kinase assay using GST-WT-HOOK3 as a substrate. NCL - No Cell Lysate control. (E) Representative autoradiogram (upper) and blot (lower) of ERK1c *in vitro* kinase assay using HOOK3 as a substrate. HeLa cells were transfected with HA-ERK1c and were treated with TPA (250 nM, 15 min), nocodazole (Noc, 100 ng/mL, 16 hr) or left non-treated (N/T). The cells were treated as described above and subjected to an *in vitro* kinase assay using GST-WT-HOOK3 as a substrate. (F) Representative autoradiogram (upper) and blots (lower) of ERK1c *in vitro* kinase assay using different HOOK3 mutations as a substrate. HeLa cells were transfected with HA-ERK1c and were treated with 100 ng/mL nocodazole for 16 hr (+) or left non-treated (−). Cells were treated as described above and then subjected to an *in vitro* kinase assay using GST-HOOK3-WT (WT), Ser162Ala (S162A), Ser238Ala (S238A) or the double HOOK3 mutant Ser162,238Ala (2SA) as a substrate. Last lane (NCL, control) contains recombinant GST-WT HOOK3 and IP of NCL (no kinase IPed). (G) Representative blots of IPed HOOK3 from HeLa cells, which were treated with nocodazole (NOC) in the presence of a MEK inhibitor. HeLa cells were transfected with GFP-HOOK3 and were either left non-treated (N/T) or treated with nocodazole (Noc, 100 ng/mL, 16 hr) with or without MEK inhibitor (PD, PD184352, 2μM, for the indicated times). Membrane was blotted with phosphorylated Ser/Thr-Pro antibody (pho-S/T-P). The bar graphs represent the average and standard errors of the intensity of phospho Ser/Thr-Pro bands in the upper panel. ∗p < 0.05 by paired t test. NRA, non-relevant antibody control; TCL, total cell lysate; IP, immunoprecipitation. All experiments, except C, were reproduced 3 times.

As HOOK3 may be essential for maintaining an intact Golgi structure, we next studied the mechanism by which it mediates ERK1c effects on Golgi fragmentation. First, we followed the expression of HOOK3 in resting and mitotic HeLa cells using Western blotting. Interestingly, we observed an upshift of the protein upon nocodazole treatment that arrests cells in prometaphase (Figure 3A), suggesting that HOOK3 might undergoes a post-translational modification during mitosis. This upshift was dependent on ERK phosphorylation, as MEK inhibitor completely reversed the nocodazole-dependent upshift (Figure 3B). Interestingly, the MEK inhibitor caused by itself an upshift of HOOK3 in cycling cells, but the identity of the involved post-translational modification or its importance was not studied. On the other hand, we further examined the nocodazole-treated HOOK3 by mass spectrometry, this analysis revealed the presence of phosphate on two Ser residues, namely Ser238 and Ser707 (Figure 3C). These sites have been previously reported in mass spectrometry screening for mitotic-specific phospho-sites but the identity of the kinase(s) that induce them, and their functions remained elusive (Dephoure et al., 2008; Olsen et al., 2010). Examination of the consensus phosphorylation sites hinted that Ser238 is phosphorylated by Pro-directed kinase that is active at mitosis. Because of the Pro at position −2, this kinase is probably one of the MAPKs (Gonzalez et al., 1991), rather than CDK, that aside of the Pro at position +1, usually requires a proximal basic amino acid and disfavor an additional nearby Pro (Chang et al., 2007). Ser707 has an Aurora consensus phosphorylation site (Ferrari et al., 2005), and is likely phosphorylated by a kinase from that family during mitosis.

As HOOK3 is a Golgi-localized protein and its phosphorylation occurs during mitosis, we then set out to confirm that ERK1c is the ERK protein that phosphorylates HOOK3 during mitosis. For this purpose, HA-ERK1 or HA-ERK1c were immunoprecipitated from resting or nocodazole-treated cells and used for an *in vitro* phosphorylation of GST-WT-HOOK3. Indeed, ERK1c that was derived from mitotic cells, but not from cells in interphase (cycling cells), significantly phosphorylated HOOK3 (Figure 3D). The phosphorylation by ERK1 from both cycling and mitotic cells was much lower than that by ERK1c, although we confirmed that both kinases from the mitotic cells were similarly active (data not shown). The specificity of the *in vitro* phosphorylation reaction was confirmed by the fact that HOOK3 was phosphorylated only by mitotic ERK1c and not by ERK1c from TPA-treated cells, in which the kinase is significantly less activated ((Shaul et al., 2009); Figure 3E). Interestingly, we noticed an additional minimal ERK consensus site within HOOK3, at Ser162, which was not detected by the mass spectrometry analysis. To reveal whether this additional site is also phosphorylated by ERK1c, we mutated both sites of ERK1c separately (S162A and S238A) and also established a doubly non-phosphorylatable mutant (S162,238A (2SA)) of the protein. Comparing the phosphorylation of the constructs by active ERK1c, we found that both WT-HOOK3 and the S162A mutant from transfected HeLa cells were phosphorylated by the active ERK1c, while the phosphorylation of S238A or 2SA was completely abolished (Figure 3F). These results indicate that HOOK3 is phosphorylated by ERK1c from nocodazole-treated cells on residue Ser238 only. Finally, we confirmed the involvement of ERK in phosphorylating the endogenous HOOK3 using anti phosphorylated Pro-Ser/Thr antibody. As expected, HOOK3 phosphorylation at this site was elevated by nocodazole treatment, and abolished in the presence of a specific MEK inhibitor for either 2 or 16 hr (Figure 3G). Taken together, our results indicate that Ser238-HOOK3 phosphorylation, which was identified by mass spectrometry, is indeed mediated by ERK1c in mitotic cells.

### ERK1c interacts with HOOK3 in the Golgi during mitosis

**Figure 4:**
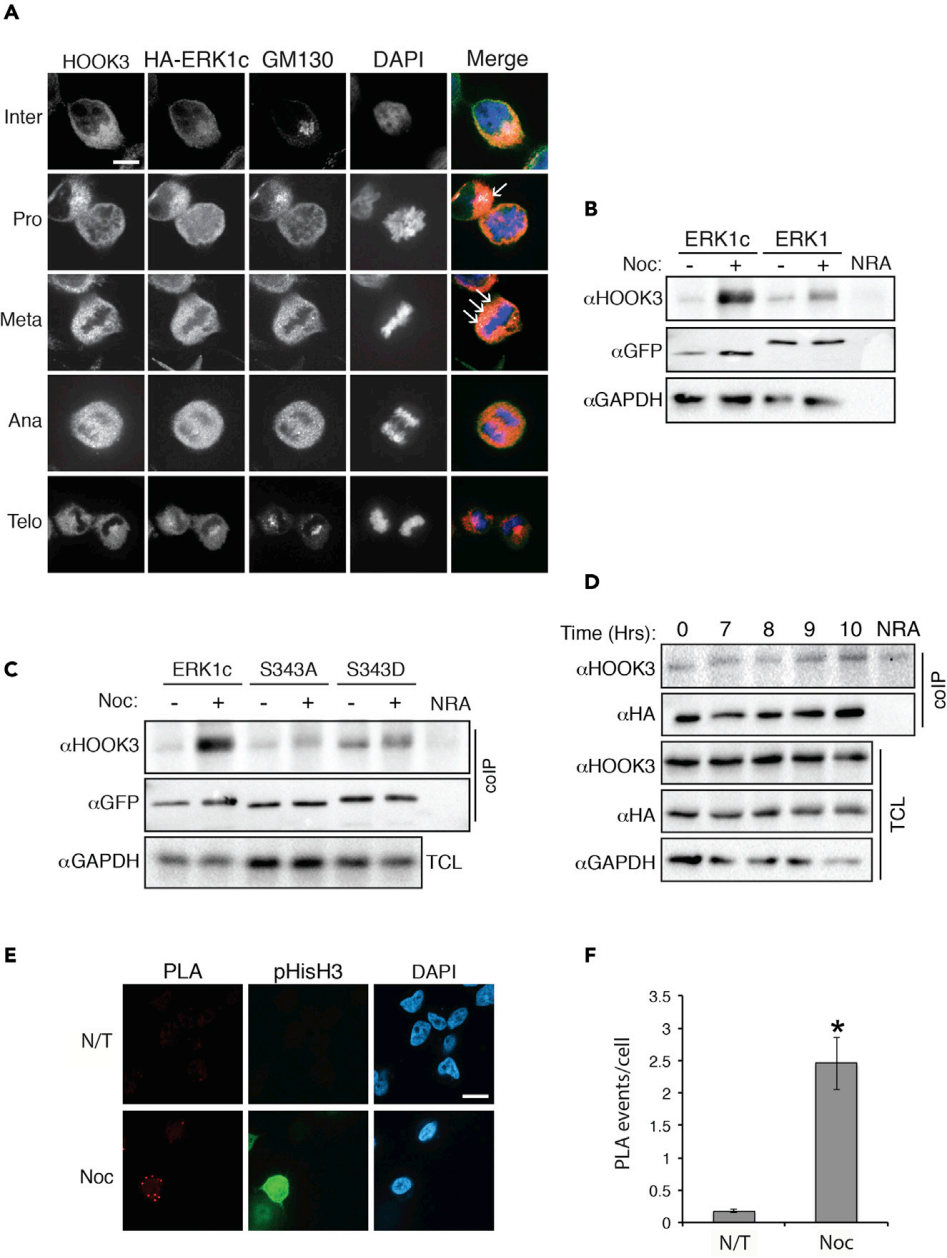
ERK1c interacts with HOOK3 in the Golgi during mitosis (A) Representative images of fluorescence microscopy of HOOK3 (pink in the merge), HA (green), GM130 (red) and DAPI (blue) staining in synchronized HeLa cells transfected with HA-ERK1c. Cells were fixed at G1/S border (Inter) and at the peak of mitosis. Cells from prophase/prometaphase (Pro), metaphase (Meta), anaphase (Ana), and telophase (Telo) were selected according to their DNA structure. Scale bar, 10μm. Arrows indicate staining's colocalization. (B) Representative blots of coIP of ERK1c or ERK1 with HOOK3 in HeLa cells transfected with GFP-ERK1 or GFP-ERK1c which were treated with 100 ng/mL nocodazole for 16 hr (+) or left non-treated (−). NRA, non-relevant antibody control. (C) Representative blots of coIP of ERK1c WT or mutated form with endogenous HOOK3 in HeLa cells transfected with HOOK3 and WT GFP-ERK1c, as well as with the non-phosphorylatable (Ser343Ala, SA) or phosphomimetic (Ser343Asp, SD) ERK1c mutants. Cells were treated with 100 ng/mL nocodazole for 16 hr (+) or left non-treated (−). TCL, total cell lysate; coIP, coimmunoprecipitation. (D) Representative blots of coIP of ERK1c with HOOK3 in HeLa cells transfected with HA-ERK1c. Cells were arrested at S-phase with thymidine following their release from the block for the indicated times. (E and F) (E) Representative images (F) and quantification of PLA analysis using anti-HA and HOOK3 antibodies of HeLa cells transfected with HA-ERK1c which were treated with 100 ng/mL nocodazole for 16 hr (Noc) or left non-treated (Con). Staining with anti (pHisH3) antibody confirmed the mitotic stage of the cells. The quantification was done as described under STAR Methods. ∗p < 0.05 by t test indicating a significant interaction as compared to control cells. Scale bar, 15μm. All experiments were reproduced 3 times. N/T, not treated.

It is well-established that ERK1/2 interacts with their substrates to confer specificity using several distinct docking domains, including D (R/KR/KX_2-6_ΦXΦ (Φ-L/I/V)) and DEF (FXFP) motifs (Yoon and Seger, 2006). HOOK3 does not have these exact sequences but does contain other sequences that resemble them (e.g. RRHLQL, three amino acid C terminals to the phosphorylation site), and may induce a MAPK interaction. We therefore undertook to study whether HOOK3 indeed interacts with ERK1c, which would further support the specific ERK1c phosphorylation. For this purpose, we first followed the subcellular localization of ERK1c and HOOK3 during interphase and mitosis to confirm their co-localization, using GM130 as a Golgi marker. Overall, we observed co-staining of ERK1c with HOOK3 in the Golgi mainly in early stages of mitosis (Figure 4A). More specifically, as expected (Wortzel et al., 2015) in cycling cells (Inter), ERK1c expression was low and mostly diffused in the cytoplasm and had no significant colocalization with HOOK3 that was observed in the cytoplasm and the Golgi. However, at prophase/prometaphase, and to some extent also metaphase, ERK1c changed its distribution, and colocalized with HOOK3 and GM130 within nuclear envelop adjacent organelle or among the spread metaphase-vesicles (white colors in merge, see arrows). No significant colocalization was observed at anaphase and in telophase, indicating that the proteins are dissociated from the Golgi at these stages. The well-established Golgi translocation of ERK1c (Wortzel et al., 2015) suggests that the organelle where the proteins are colocalized is the fragmented Golgi apparatus (at least in prophase/prometaphase). However, as the diffused nature of the staining could be incidental, and colocalization do not necessarily indicate a direct binding, we resorted to more specific techniques to study the interaction.

We then examined whether ERK1c and HOOK3 can directly interact with each other. To this end, we transfected HeLa cells with GFP-ERK1 or GFP-ERK1c, followed by a nocodazole treatment. Using co-immunoprecipitation (coIP), we found that HOOK3-ERK1c interaction is increased in mitosis, and this interaction was considerably stronger than with ERK1 (Figure 4B). The differences in HOOK3 interaction might be due to the mitotic ERK1c shuttling to the Golgi, which is regulated by Ser343-ERK1c phosphorylation by CDK1 (Wortzel et al., 2015). In order to confirm that Golgi localization of ERK1c is required for the interaction and phosphorylation of HOOK3, we used mutants of the Ser343-ERK1c with a modulated capacity to translocate to the Golgi (Wortzel et al., 2015). Thus, we transfected HeLa cells with either WT-ERK1c, non-phosphorylatable (S343A), or phosphomimetic (S343D) ERK1c mutants, and determined their interactions with HOOK3. As expected, the WT-ERK1c interacted weakly with HOOK3 in cycling cells, and this interaction increased notably upon nocodazole treatment (Figure 4C). The S343A mutation, which fails to translocate to the Golgi, reduced the interaction in both cycling and mitotic cells. On the other hand, the S343D mutation increased the interaction in cycling cells to some extent, without much change on the interaction upon nocodazole treatment. This result is in line with the effect of this mutant on Golgi translocation and the activity of ERK1C (Figure 4 and (Wortzel et al., 2015)). These results indicate that the mitotic interaction and phosphorylation of HOOK3 by ERK1c occurs in the Golgi, and requires the shuttling of ERK1c into this organelle.

To further confirm the ERK1c-HOOK3 interaction in mitosis, we transfected cells with HA-ERK1c, arrested them at the beginning of S-phase with thymidine, and then released them from the block for the indicated times, knowing that mitosis occurs at 9-10 hr after release. The ERK1c-HOOK3 interaction was then determined by coIP that showed an increase toward mitosis, reaching a peak at 9-10 hr (Figure 4D), thus confirming that the interaction is dependent on mitosis. An additional indication for ERK1c-HOOK3 interaction was observed by proximity ligation assay (PLA), in which HA-ERK1c overexpressing cells treated with nocodazole showed significant staining as compared with non-treated cells (Figures 4E and 4F). Staining with anti (pHisH3) antibody confirmed the mitotic stage of the cells. Taken together, our results clearly indicate that ERK1c, and not ERK1, interacts with HOOK3 during mitosis, confirming the specificity of Ser238 phosphorylation by ERK1c.

### HOOK3 is phosphorylated by AuroraA

**Figure 5:**
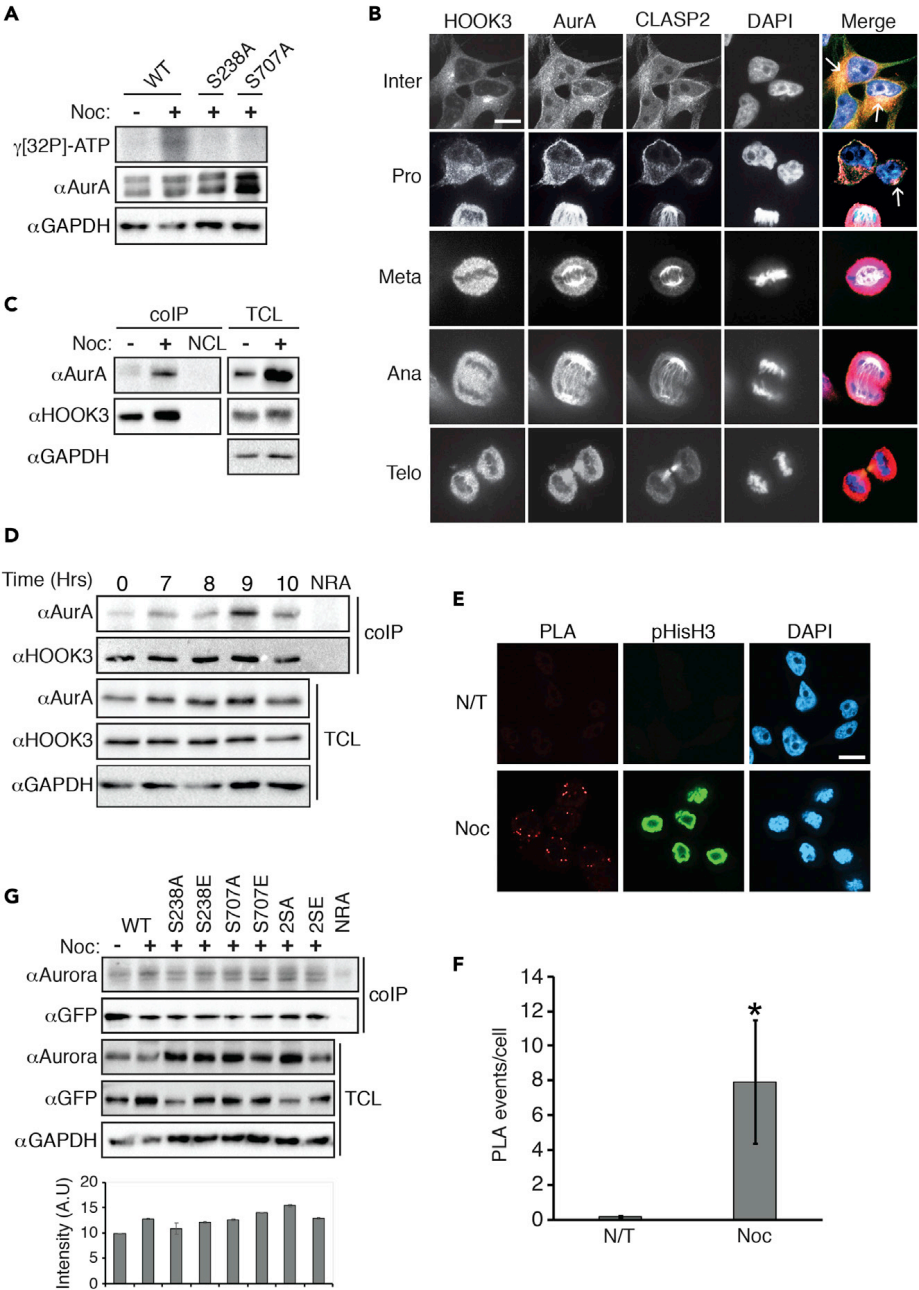
HOOK3 is phosphorylated by AuroraA during mitosis (A) Representative autoradiogram (upper) and blot (lower) of AuroraA *in vitro* kinase assay using HOOK3 as a substrate. Endogenous AuroraA was immunoprecipitated from HeLa cells which were treated with 100 ng/mL nocodazole for 16 hr (+) or left non-treated (−). The IPed kinase was then subjected to an *in vitro* kinase assay using GST-WT-HOOK3 as a substrate or its non-phosphorylated mutants, S238A or Ser707Ala (S707A). (B) Representative images of fluorescence microscopy of HOOK3 (pink in the merge), AuroraA (AurA, red), CLASP2 (green) and DAPI (blue) staining in synchronized HeLa cells. Cells were fixed at G1/S border (Inter) and at the peak of mitosis. Cells from prophase/prometaphase (Pro), metaphase (Meta), anaphase (Ana), and telophase (Telo) were selected according to their DNA structure. Scale bar, 10μm. Arrows indicate staining's colocalization. (C) Representative blots of coIP of endogenous AuroraA with HOOK3 in HeLa cells which were treated with 100 ng/mL nocodazole for 16 hr (+) or left non-treated (−). coIP, coimmunoprecipitation; TCL, total cell lysate. (D) Representative blots of coIP of endogenous AuroraA with HOOK3 in HeLa cells. Cells were arrested at G1/S border by the double thymidine block, followed by a release from the block for the indicated times. NRA, non-relevant antibody control. (E and F) Representative images (E) and quantification (F) of PLA analysis using anti-AuroraA and HOOK3 antibodies of HeLa cells which were treated with 100 ng/mL nocodazole for 16 hr (Noc) or left non-treated (N/T). Staining with anti (pHisH3) antibody confirmed the mitotic stage of the cells. The quantification was done as described under STAR Methods. Scale bar, 15μm. (G) Representative blots of coIP of AuroraA with HOOK3 in HeLa cells transfected with GFP WT-HOOK3 or its non-phosphorylated or phosphomimetic mutants on either ERK1c site (S238A, S238E), AuroraA site (S707A, S707E), or both phosphorylated residues (2SA, 2SE). Cells were treated with 100 ng/mL nocodazole for 16 hr (+) or left non-treated (−). The Bar graph represents the average and standard errors of the intensity of AuroraA bound to HOOK3 (upper panel). No statistically significant changes were observed. All experiments were reproduced 3 times.

Apart from the ERK1c phosphorylation site, the mass spectrometry analysis revealed the existence of phosphate also on Ser707 (Figure 3C) that is located within the consensus phosphorylation site of Aurora family of kinases (Ferrari et al., 2005). Aurora kinases are known to play key roles in mitosis, and of the three family members, AuroraA is the one that is mostly related to the regulation of Golgi architecture (Kimura et al., 2018). Therefore, we undertook to determine the role of this kinase in HOOK3 phosphorylation during mitosis. To this end, we immunoprecipitated AuroraA from HeLa cells that were either treated with nocodazole or left untreated, and incubated the IPed samples with GST-WT-HOOK3 or its non-phosphorylatable mutants, S238A or Ser707Ala (S707A), in the presence of γ[^32^P]-ATP. We found that indeed, AuroraA from mitotic cells phosphorylated WT-HOOK3, but not the S707A mutant, confirming this residue as the phosphorylated site (Figure 5A). Interestingly, the S238A-HOOK3, which is unable to be phosphorylated by ERK1c, but has intact Ser707, was not phosphorylated as well. This may suggest that the phosphorylation of Ser238 by ERK1c is a pre-requisite for its further phosphorylation on Ser707 by AuroraA.

As for ERK1c, we next undertook to examine whether AuroraA interacts with HOOK3, which would provide additional support for the specific phosphorylation. First, we co-stained HOOK3 and AuroraA in interphase and at the different stages of mitosis in HeLa cells. CLASP2, which interacts with the Golgi and MTs in interphase but detaches from the Golgi and remain attached to the MTs in mitosis (Liu et al., 2007), was used as a MTs localization marker. Indeed, we observed co-staining of HOOK3 with AuroraA and CLASP2 in interphase but much less during mitosis (Figure 5B). More specifically, HOOK3 was mostly localized in the Golgi of cycling cells (Inter), while AuroraA was detected in both the cytoplasm and the Golgi of HeLa cells, where the three proteins colocalized (white color, arrows in merge panels). At prophase/prometaphase the colocalization was much less clear (arrow in the merge panel), probably because of the detachment of CLASP2, but a possible colocalization of AuroraA with HOOK3 was still visible. In metaphase and anaphase, there was no apparent colocalization, as HOOK3 was localized with the Golgi vesicles, while AuroraA was mostly colocalized with the CLASP2 containing MTs. In telophase, where the new Golgi is already rebuilt in the two daughter cells, HOOK3 and AuroraA were colocalized, probably in the regenerated Golgi, but no co-staining was seen with the MTs/CLASP2 at this stage (Figure 5B).

This colocalization of HOOK3 and AuroraA prompted us to further examine whether the two proteins directly interact with each other. For this purpose, we first used coIP of AuroraA with HOOK3, and found a direct interaction between them in nocodazole-treated HeLa cells (Figure 5C). Then, we extracted the AuroraA and HOOK3 from cells released from a thymidine block and found that similarly to the interaction with ERK1c, a specific interaction of HOOK3 with AuroraA peaked 9-10 hr after release (Figure 5D). An additional indication for AuroraA-HOOK3 interaction was observed by PLA assay, in which HeLa cells treated with nocodazole showed a significant number of interactions between the endogenous proteins (Figures 5E and 5F). Staining of phosphorylated histone (pHisH3) confirmed that the interaction occurs in mitosis. Finally, we wanted to examine whether AuroraA and HOOK3 interact when the latter is already phosphorylated either by ERK1c, by AuroraA, or both. Therefore, we overexpressed GFP-WT-HOOK3 or its non-phosphorylatable or phosphomimetic mutants of either ERK1c site (S238A, S238E), AuroraA site (S707A, S707E), or both phosphorylated residues (2SA, 2SE). Then, the cells were either left untreated or treated with nocodazole, harvested, and subjected to coIP. A clear AuroraA-WT-HOOK3 interaction was observed in extracts of cycling cells, and this was slightly increased after nocodazole treatment (Figure 5G). Furthermore, a significant interaction with AuroraA was observed in all phosphorylation sites mutants treated with nocodazole (Figure 5G), indicating that the phosphorylations are probably not important for the HOOK3-AuroraA interaction. Therefore, the increase in HOOK3-AuroraA interaction upon nocodazole treatment might be due to elevated expression or mitotic modifications of AuroraA, or Ser238/Ser707 phosphorylation-independent post-translational modifications of HOOK3. Taken together, the results indicate that AuroraA is colocalized with CLASP2/MTs throughout the cell cycle, but some of its molecules interact with HOOK3 in prophase/prometaphase, to allow phosphorylation of S707. This interaction is not dependent on the phosphorylation of HOOK3 by ERK1c.

### HOOK3 interaction with MTs is disrupted due to its mitotic phosphorylations

**Figure 6:**
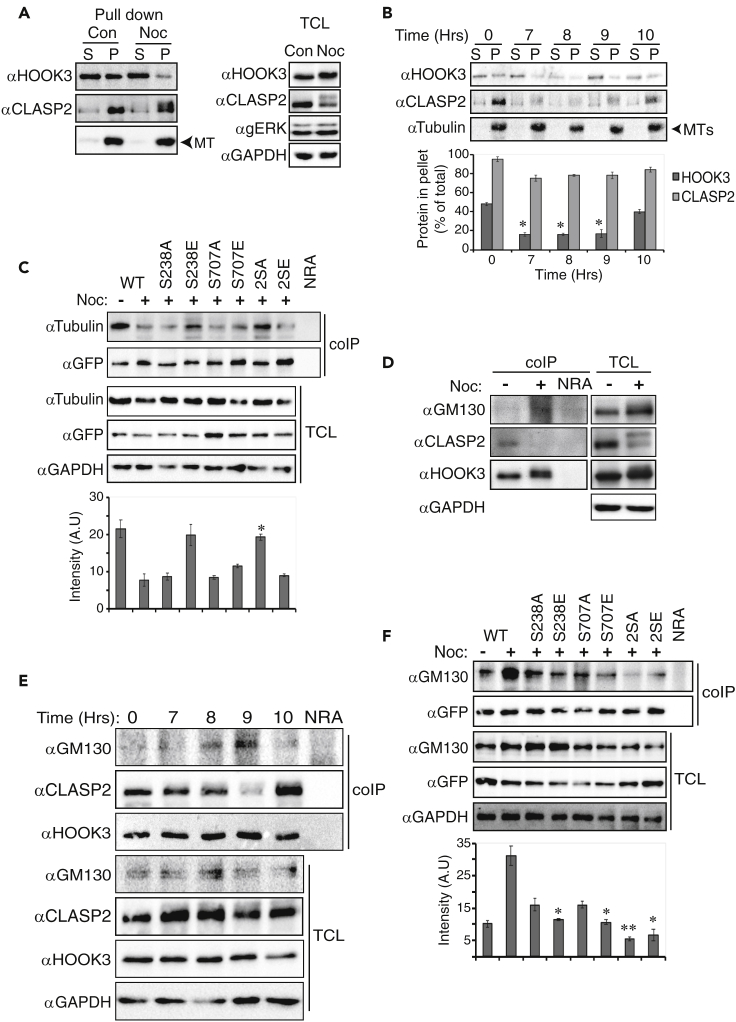
HOOK3 interaction with microtubules is disrupted by its mitotic phosphorylations (A) Representative blots of microtubule-associated protein spin-down assay which was performed according to the manufacturer's instructions using HeLa cells. Cells were treated with 100 ng/mL nocodazole for 16 hr (Noc) or left non-treated (N/T) and supernatant (S) and pellet (P) fractions are indicated, as well as total cell lysates (TCLs). (B) Representative blots of microtubule-associated protein spin-down assay as described above which was performed on HeLa cells that were arrested at a G1/S border by the double thymidine block, followed by a release from the block for the indicated times. S, supernatant; P, pellet; MTs, microtubules. The bar graph represents average and standard errors of percent protein in the pellet out of the total protein for HOOK3 (dark gray) and CLASP2 (light gray) at the indicated timepoints. It should be noted that HOOK3 and CLASP2 recognition by their antibodies varies between the different time points due to the phosphorylated states of the proteins. Thus, quantification was calculated as described above taking into consideration all relevant bands. ∗p < 0.05 by paired t test. (C) Representative blots of coIP of tubulin with HOOK3 in HeLa cells transfected with GFP WT-HOOK3 or its non-phosphorylated or phosphomimetic mutants on either ERK1c site (S238A, S238E), AuroraA site (S707A, S707E), or both phosphorylated residues (2SA, 2SE). Cells were treated with 100 ng/mL nocodazole for 16 hr (+) or left non-treated (−). The bar graph represents the average and standard errors of the intensity of tubulin bound to HOOK3 (upper panel) ∗p < 0.05 by paired t test. coIP, coimmunoprecipitation; TCL, total cell lysate; NRA, non-relevant antibody. (D) Representative blots of coIP of HOOK3 with GM130 or CLASP2 in HeLa cells which were treated with 100 ng/mL nocodazole for 16 hr (+) or left non-treated (−). (E) Representative blots of coIP of GM130 or CLASP2 with HOOK3 in HeLa cells transfected with GFP WT-HOOK3 and arrested at S-phase with thymidine following their release from the block for the indicated times. This is the same coIP experiment shown in Figure 5D that was reblotted with GM130 and CLASP2. Therefore, the panels of HOOK3 IP and HOOK3 and GAPDH of TCL are the same. (F) Representative blots of coIP of GM130 with HOOK3 in HeLa cells transfected with GFP WT-HOOK3 or its non-phosphorylated or phosphomimetic mutants on either ERK1c site (S238A, S238E), AuroraA site (S707A, S707E), or both phosphorylated residues (2SA, 2SE). Cells were treated with 100 ng/mL nocodazole for 16 hr (+) or left non-treated (−). The bar graph represents the average and standard errors of the intensity of GM130 bound to HOOK3 (upper panel). ∗p < 0.05, ∗∗p < 0.01 by paired t test. All experiments were reproduced 3 times.

Next, we were interested to study how HOOK3 phosphorylation contributes to mitotic Golgi fragmentation. HOOK3 was proposed in the past to interact not only with the Golgi apparatus but also with MTs (Walenta et al., 2001), which may play a role in the stabilization of the Golgi apparatus in interphase. Therefore, we hypothesized that HOOK3 interaction with MTs is disrupted during mitosis due to HOOK3 phosphorylation. To address this, we used the MTs-associated protein spin-down assay that gives information about the ability of proteins to bind MTs. Thus, when a lysate from cycling cells was incubated with the MTs, HOOK3 was found in equal levels in both the pellet and the supernatant. This result confirms the previous observations that HOOK3 has the ability to interact with the MTs in cycling cells (Figure 6A). However, almost no HOOK3 was detected in the pellet of nocodazole-treated cells, indicating that HOOK3 lost its ability to interact with MTs during mitosis. Moreover, when tested in cells that were released from a double thymidine block, this interaction was significantly diminished at the peak of mitosis (9-10 hr Figure 6B). On the other hand, the MTs interaction with the other Golgi-MTs bridging protein, CLASP2 was not changed during mitosis (Figures 6A and 6B). This indicates that while CLASP2 constantly binds to MTs throughout the cell cycle, HOOK3 detaches from it during mitosis.

Upon showing that the ability of HOOK3 to interact with MTs depends on mitosis, we further aimed to show that it is actually the switch of HOOK3 phosphorylation status which determines this interaction. To this end, we first overexpressed GFP-WT-HOOK3, treated the cells with nocodazole, and subjected their extracts to a coIP using HOOK3 antibody that precipitates both exogenous and endogenous proteins. Interestingly, we found that the overexpression interferes with endogenous HOOK3-MTs interaction in nocodazole treated, but not interphase cells (Figure S4). This may indicate that excess of phosphorylated protein (nocodazole-treated), but not non-phosphorylated one (interphase) competes with the MTs-HOOK3 interaction. Therefore, we examined the effect of HOOK3 phosphorylation on the binding to MTs by using phosphorylation site mutants. It should be noted that in this experiment we assessed the interaction of the exogenous protein only, which is independent from the reduction seen with the endogenous proteins in Figure S3. Similarly to the experiments above, a clear interaction was observed between HOOK3 and tubulin in cycling cells, while this interaction was dramatically reduced upon nocodazole treatment (Figure 6C). Most mono-phosphorylation sites mutants had only minor effects on the mitotic interaction, except of S238E that showed some increased interaction, albeit not statistically significant. The only significant effect was observed with the 2SA mutation, which reversed the reduced interaction, bringing it back to the level of interaction in cycling cells. Together, these results indicate that both phosphorylations are required for the mitotic detachment of HOOK3 from the MTs, while each one of them alone seems to have very little effect. Although the coIP may reveal interaction with free tubulin and not only MTs, the reduced interaction in mitosis clearly indicates that the interaction with any of these tubulin pools is prevented.

### HOOK3 interaction with GM130 in the Golgi is increased in mitosis

As HOOK3 no longer interacts with the MTs but maintained its association with the fragmented Golgi, we next examined the interaction of HOOK3 with the Golgi structural protein GM130 (Barr and Short, 2003) in cycling, as well as mitotic cells. The coIP showed no significant interaction between these proteins in cycling cells, while the MTs binding protein CLASP2 did interact with HOOK3 (Figures 6D and 6E). The fact that HOOK3 does not interact with GM130 but does interact with MTs in interphase, along with previous finding that GM130 is responsible for MTs nucleation in the Golgi in interphase (Millarte and Farhan, 2012), suggest that HOOK3 (together with CLASP2) is responsible for linking MTs that originates out of the Golgi, and not those nucleated in the Golgi. Importantly, these interactions were reversed in mitosis: while the HOOK3-CLASP2 interaction was significantly decreased, the interaction with GM130 was increased upon nocodazole treatment (Figure 6D), or in peak mitosis upon release from double thymidine block (Figure 6E). We also validated the mitotic interaction between endogenous HOOK3 and GM130 using PLA (Figure S5A). These findings indicate that while MTs-bound CLASP2 is dissociated from HOOK3, the interaction of HOOK3 with the fragmented Golgi is increased in mitosis. We then studied the effect of phosphorylation on the HOOK3-GM130 interaction and found that the interaction was reduced by alanine substitution of the mono-phosphorylated sites, and the double alanine mutant (2SA) significantly abolished it (Figure 6F). Interestingly, the interaction of the phosphomimetic substitutions with GM130 (particularly 2SE) was not increased. This might be due to the fact that the acidic amino acids do not imitate the phosphorylated residue as occurs also in few other cases (e.g. E3-ligases (Rubio de la Torre et al., 2009)), or because these mutants do not play a dominant positive role over the endogenous proteins. However, despite the lack of phosphomimetic mutant effects, the results with the Ala mutants are compelling and sufficient to show that both phosphorylations are required for HOOK3 interaction with GM130 and its release from MTs.

Since we observed distinct interactions with MTs throughout the cell cycle, we further studied this phenomenon. For this purpose, we stained HOOK3, CLASP2, and tubulin to follow their distribution in cycling and mitotic HeLa cells. As expected, HOOK3 and CLASP2 were localized in the Golgi of cycling cells, while MTs were seen all over the cell, including the Golgi (Inter, Figure S5B, white color and arrow in the merge panel). To further study this point, we co-stained tubulin and HOOK3 only (Figure S6A), and found that these two proteins colocalize all over the cell, and not particularly in the Golgi in interphase. On the other hand, the MTs interactor CLASP2 demonstrated a clear Golgi localization in interphase, while it was mostly absent from the Golgi during mitosis (Figures S5B and S6B). Thus, we concluded that at interphase, HOOK3 can interact with MTs in the Golgi, but also in other areas of the cell. At the onset of mitosis, HOOK3 remains colocalized with the fragmented Golgi, while CLASP2 is found colocalized with the MTs. Additionally, when HOOK3 and CLASP2 colocalization was analyzed by imaging flow cytometry (Figure S7), we found that these proteins share a high bright detail similarity (BDS), which indicates a colocalization during interphase (data not shown) and G2. In prophase, more than half of the population demonstrated a reduction in the BDS value, signifying the beginning of separation, which was completed during metaphase-telophase, in which the BDS value is low. These results are in agreement with the results obtained for the interaction between these proteins and suggest that these dynamic changes play a role in the destabilization of the Golgi and initiation of its fragmentation.

### HOOK3 phosphorylation derives mitotic progression

**Figure 7:**
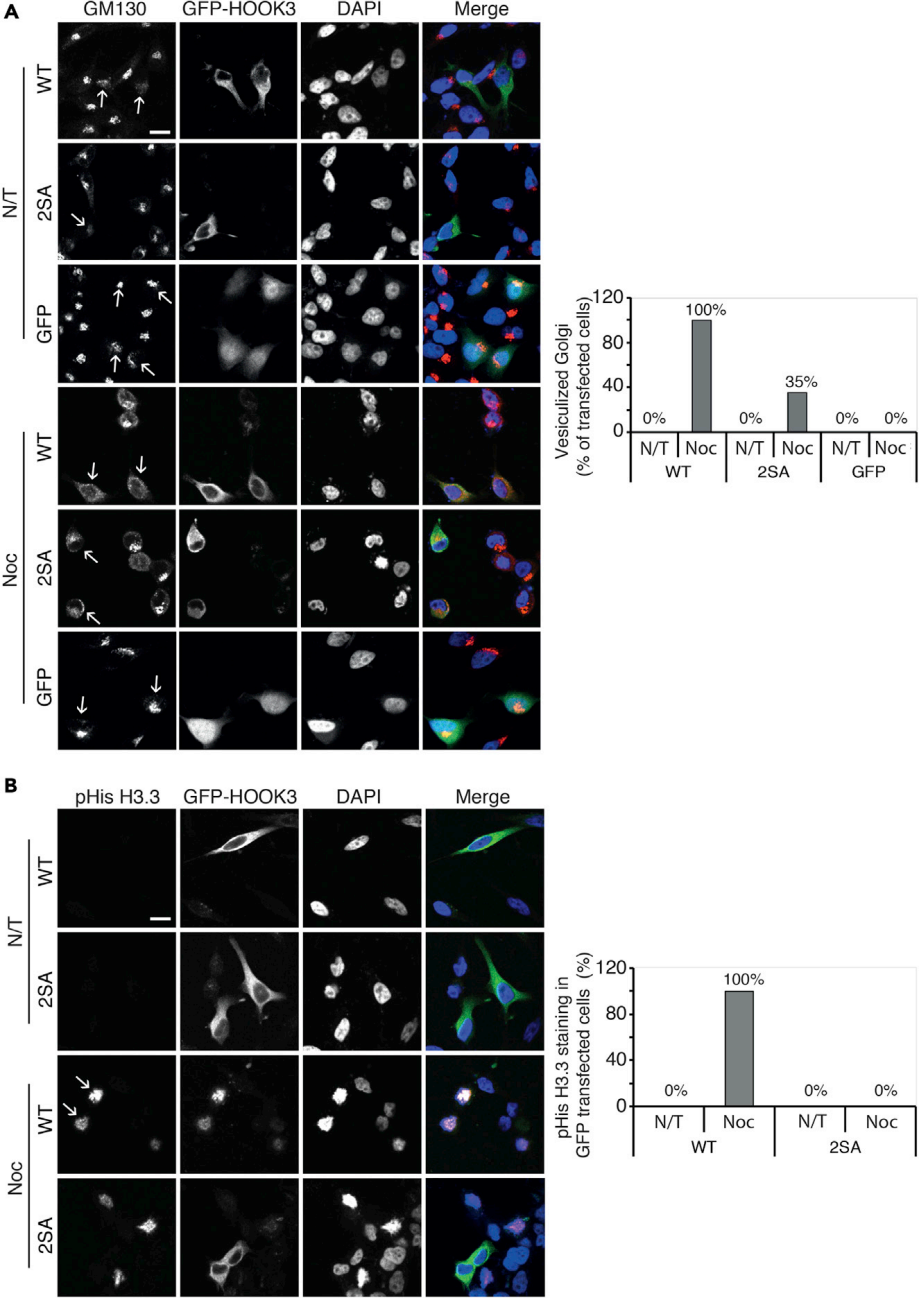
HOOK3 phosphorylation derives mitotic progression (A) Representative images (left) and quantification (right) of fluorescence microscopy of GM130 (red), GFP-HOOK3 (green) and DAPI (blue) staining in HeLa cells. Cells were transfected with WT-HOOK3 (WT), non-phosphorylatable HOOK3 mutant (S238,707A, 2SA) or with GFP only (GFP), and treated with 100 ng/mL nocodazole for 16 hr (NOC) or left non-treated (N/T). Quantification of cells with vesiculated Golgi (bar graph at right) was done on cells containing GFP positive stain (transfected cells). Scale bar, 10μm. (B) Representative images (left) and quantification (right) of fluorescence microscopy of pHis H3.3 S31P (red), GFP-HOOK3 (green), and DAPI (blue) staining of HeLa cells which were treated as described in panel (A). Quantification of cells stained for pHis3.3 (bar graph at right) was done on cells containing GFP-positive stain (transfected cells). Scale bar, 10μm. Arrows indicate staining in transfected cells.

Upon showing that HOOK3 phosphorylation by ERK1c and AuroraA switches its interaction from MTs to GM130, it was important to study the direct effect of these phosphorylations on Golgi fragmentation and cell cycle progression. Therefore, we followed the changes in the Golgi architecture in cells expressing WT or 2SA mutant (Figure 7A). Similarly to a previous study (Walenta et al., 2001), overexpression of HOOK3 induced some Golgi chunk formation, which resembled initial steps of mitotic fragmentation. This effect was variable among the cells, and was not seen in all experiments. However, overexpression of the 2SA mutant had no effect on the interphase Golgi structure, and as we showed above (Figure 6C), it did not change MTs-HOOK3 interaction as well. Therefore, we concluded that the small amount of fragmentation detected is not related to the HOOK3 effects described above. On the other hand, overexpression of WT-HOOK3 induced significant Golgi vesiculation in nocodazole-treated cells that resembled Golgi appearance in metaphase. This was a different phenotype from the typical prometaphase-like partial chunk formation that was observed in the nocodazole-treated cells that did not contain overexpressed HOOK3 (GFP only or the non-transfected cells; Figure 7A). This effect correlated with the effect of overexpressed HOOK3 on MTs-HOOK3 interaction in nocodazole-treated cells (Figure S4), suggesting that the reduced HOOK3-MTs interaction is the mechanism that allows the progression of Golgi fragmentation to the vesiculation stage. Importantly, this effect was completely reversed by the 2SA mutation, showing the importance of HOOK3 phosphorylation in driving Golgi fragmentation.

Since phosphorylation of HOOK3 is necessary for a metaphase-like structure of the Golgi, without affecting the DNA reorganization at these stages, we undertook to find out the effect of HOOK3 phosphorylation on another aspect of mitotic progression. To this end, we followed the phosphorylation of Histone H3.3 on Ser31 in the presence of overexpressed WT or 2SA mutant HOOK3. The phosphorylation of Histone H3.3 on S31 appears in late prometaphase and metaphase, and therefore can be used as a metaphase marker (Hake et al., 2005), unlike histone 3 (Figure 4) that serves as a general mitotic marker. As expected, no S31-H3.3 staining was apparent in cycling cells regardless of the overexpression (Figure 7B). However, while there was a significant S31-H3.3 staining in WT-HOOK3 transfected cells treated with nocodazole, cells expressing the 2SA mutant were not stained at all (Figure 7B). These results indicate that enhanced Golgi fragmentation drives additional metaphase-related processes, although not all of them (e.g. DNA rearrangement). In summary, the phosphorylations of HOOK3 by ERK1c and AuroraA at the onset of mitosis are essential for Golgi fragmentation and mitotic progression.

## Discussion

**Figure 8:**
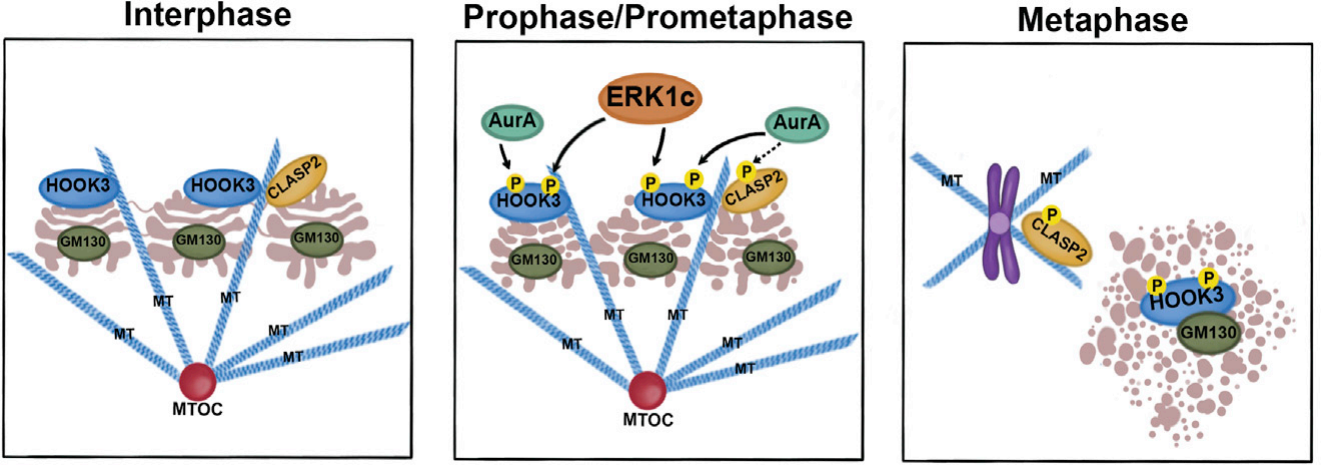
Schematic presentation of ERK1c-HOOK3 function An illustration demonstrating the role of ERK1c, AuroraA (AurA), microtubules (MT), HOOK3, GM130 and CLASP2 in relation to the Golgi fragmentation process and HOOK3 phosphorylations at different stages of the cell cycle. Interphase: During interphase the Golgi is organized in stacks, while GM130 is found in between these stacks. The MTs are being polymerized from the microtubules originating center (MTOC) and stabilizing the Golgi structure. Both HOOK3 and CLASP2 interact together with the MTs and Golgi. Prophase/Prometaphase: During the entry to mitosis, HOOK3 is being phosphorylated by both ERK1c and AurA, while CLASP2 might be phosphorylated by AurA. At this time the Golgi stacks starts to break into ribbons. Metaphase: The phosphorylation of HOOK3 and CLASP2 allow the complete fragmentation of the Golgi into a haze. At this time, phosphorylated CLASP2 maintain its interaction with the MTs that originate from the centromeres, while phosphorylated HOOK3 interacts with GM130.

Major architectural changes occur within a cell during mitosis to ensure the proper separation of genetic and other cellular materials between daughter cells (Champion et al., 2017). One of the cellular organelle that we were interested in is the Golgi apparatus, which undergoes an extensive disassembly (fragmentation) at the onset of mitosis and is rebuilt at the end of mitosis to form a full apparatus in each of the daughter cells (Colanzi et al., 2003). In this paper, we have demonstrated and characterized the involvement of HOOK3 in the process of Golgi fragmentation, and have shown that it is tightly regulated via phosphorylation by the protein kinases ERK1c and AuroraA. Overall, our findings best fit a model in which HOOK3 serves as a regulator of Golgi localization and stabilization during interphase, by linking the Golgi to the MTs network (Figure 8A) originating out of the Golgi. This interaction seems to be accompanied, and probably strengthened, by CLASP2, thereby showing that both bridging proteins play a role in Golgi stabilization during interphase. This is supported by the fact that knockout of each one of them is sufficient to induce a massive fragmentation ((Miller et al., 2009) and Figure 2). During prophase, when Golgi fragmentation is initiated, ERK1c phosphorylates HOOK3 on Ser238 that facilitates an additional phosphorylation on Ser707 by AuroraA (Figure 8B). This post-translational modification triggers the detachment of phosphorylated HOOK3 from the MTs and facilitates its binding to GM130 within the Golgi vesicles (Figure 8C). In parallel, this process is accompanied by detachment of phosphorylated CLASP2 (Deretic et al., 2019) from the Golgi and its facilitated binding to the MTs. This double detachment indicates that all MTs, irrespective to their site of nucleation, are detached from the Golgi, and this induces the destabilization and fragmentation of the Golgi at mitosis.

The Golgi complex in mammalian cells is organized as a ribbon-like array of several polarized stacks of membrane cisternae that are connected by lateral tubules (Makhoul et al., 2018). The Golgi architecture depends on members of both the GRASP and the golgin families of membrane-tethering proteins. While GRASP55 and GRASP65 act as membrane tethers that hold together the cisternae as stacks, the golgins provide an additional level of support that tether cytoskeletal components and membranes. Moreover, its association with the MTs keeps the Golgi apparatus in the perinuclear region next to the centrosome. However, during several cellular processes, the Golgi ribbon breaks and is converted into individual stacks which are further disassembled. Mitosis is the most prevalent process that requires Golgi fragmentation that in this case is required for the division of the Golgi into two daughter cells. Other processes that may involve Golgi fragmentation, mostly partial, include migration, apoptosis, neurological disorders and cancers (Caracci et al., 2019; Donizy and Marczuk, 2019; Farhan and Hsu, 2016). The mechanism that allows the fragmentation in all cases involves phosphorylation of the Golgi structural proteins, which allow its release from protein and lipid complexes and breakage of the membranes that connect the stacks together (Ayala and Colanzi, 2017; Colanzi et al., 2003). However, the regulation of this effect seems to vary between different conditions, and the exact mechanism and regulation in each case are still unclear.

In this work we characterized HOOK3 as a protein who further stabilizes the intact Golgi structure during interphase and its regulation upon mitosis to enable Golgi disassembly. The HOOKs are a family of proteins that have been originally identified due to the peculiar hooked-bristle phenotype in hook-deficient Drosophila melanogaster (Kramer and Phistry, 1996). The family of human HOOKs includes three homologs, HOOK1, HOOK2, and HOOK3 that are abundantly expressed in human cells, mainly functioning as MTs to membrane binding proteins. Therefore, the HOOKs were suggested to act as adaptor/bridging proteins involved in linking membrane compartments to MTs (Szebenyi et al., 2007; Walenta et al., 2001). Indeed, it was shown that HOOKs may participate in the trafficking of membrane vesicles and protein complexes along MTs between the Golgi apparatus, centrosomes (Baron Gaillard et al., 2011; Ge et al., 2010; Szebenyi et al., 2007; Walenta et al., 2001), endosomes (Richardson et al., 2004) and lysosomes (Xu et al., 2008). As of today, very little is known about HOOK3, although it is clear that it interacts with Golgi membranes and is enriched in the cis-Golgi *in vivo* (Walenta et al., 2001). Therefore, it is likely that the HOOK3 effect in interphase is bridging between the Golgi membranes to the MTs. The binding through Golgi membranes is supported by the fact that no significant interaction with structural Golgi proteins (GRASP55, GRASP65, or GM130) was detected in cycling cells under all conditions examined.

It was initially suggested that HOOK3 is involved in Golgi stability (Walenta et al., 2001), but these results were based on overexpression of the protein. In our study, the effect of overexpression was not so pronounced and only rarely induced fragmentation. On the other hand, reducing HOOK3 expression resulted in a substantial effect on the Golgi architecture. This indicates that the presence of this protein is essential for Golgi stabilization, and when absent, either by knockout or due to dissociating following phosphorylation, the Golgi undergoes breakdown. The effect of HOOK3 overexpression could have been due to a lack of enough binding sites on the Golgi membranes, so that the excess protein competes with the interaction of the endogenous protein with MTs, without a mutual binding to the Golgi membranes. Another possibility is that the excess protein is hyperphosphorylated, which is the reason for its competing effect on the endogenous protein. Nonetheless, this effect does not contradict our findings that HOOK3 phosphorylations negate the interaction with MTs and therefore has a similar effect to that of a knockout.

The specific phosphorylation of HOOK3 by ERK1c on Ser238 that we identified here seems to be a preceding, priming, phosphorylation for a sequential phosphorylation by AuroraA on Ser707. Both these phosphorylations are required for the detachment from MTs, and interaction with GM130, while each one of them alone seems to have only a partial effect. On the other hand, the full phosphorylation of both sites is required for the strong binding to GM130, but in this case, the single phosphorylations have very minor effects. Such priming phosphorylations that set the ground for additional phosphorylations by the same or other protein kinases are known to occur in tightly regulated systems. One example is β-Catenin that is phosphorylated first by CK1 that allows a follow-up GSK3 phosphorylation (Harwood, 2002). In addition, it also seems to fit in the phosphorylation of ERK1/2 by MEK, where the phosphorylation of the activatory Tyr residue is a prerequisite for the phosphorylation of the Thr residues (Seger and Krebs, 1995). In addition, we have previously found such a system in the regulation of ERK1c that is phosphorylated on Ser343 by CDK1, and this phosphorylation is required for the translocation to the Golgi where the protein is further phosphorylated by MEK1b (Wortzel et al., 2015). However, Ser343 cannot be considered as priming phosphorylation because MEK1b can phosphorylate ERK1c when in the Golgi even without the pSer343.

The GRASP proteins are considered the main regulators that control equilibrium between the Golgi stacking and ribbon formation, and as such they play an important role in governing the Golgi architecture. Phosphorylation of GRASP55 and GRASP65 by various protein kinases was implicated in the destabilization of the ribbons, and hence the initiation of Golgi fragmentation. Since GRASPs play such an important role in the Golgi architecture, we were interested in determining whether they may be associated with the ERK1c-HOOK3 effect of Golgi fragmentation. However, our results indicate that neither the knockdown of GRASP55 nor of GRASP65 had a significant effect on the ERK1c-induced fragmentation, although the knockdown of GRASP65 and to some extent also GRASP55 did affect fragmentation in ERK1 overexpressing cells. This result may indicate that GRASP55, and more so GRASP65, are involved in the stage at which ERK1/2 regulate the entrance to mitosis but not during the ERK1c-regulated stage that occurs later on. To confirm that GRASP65 is not involved in the ERK1c-HOOK3 axis of the fragmentation, we further studied whether GRASP65 interacts with HOOK3 or ERK1c and found no clear indication for such interaction using coIP or PLA assays, regardless of HOOK3 phosphorylation state (data not shown). As mentioned above, the fact that GRASPs, as well as GM130 do not interact with HOOK3 also indicate that the interaction is with MTs that are originated in MTOCs outside the Golgi and not in the MTs initiated in the Golgi (Miller et al., 2009).

In our study, we aimed to identify substrates by which ERK1c exerts its mitotic Golgi fragmentation. We have demonstrated and characterized the first substrate, HOOK3, its phosphorylation sites and its protein-protein interactions. We identified a novel function of HOOK3, as an essential protein for the stabilization of the intact structure of the Golgi apparatus during interphase. HOOK3 contain both MTs and Golgi localizing motifs, and thus serve as a bridge between them. The interaction of HOOK3 with the MTs is tightly regulated via phosphorylation by the protein kinases ERK1c and AuroraA. This double phosphorylation mediates the detachment of HOOK3 from the MTs, therefore undermines the Golgi structure and allows its mitotic fragmentation. However, it is clear from our findings that the process of Golgi fragmentation is regulated by more proteins and phosphorylations than was reported before (Ayala and Colanzi, 2017; Ayala et al., 2020). The identification of these processes and components requires further studies.

### Limitations of the study

The architecture and activity of the Golgi apparatus is well regulated by several processes, including phosphorylation (Huang and Wang, 2017), which is mediated in part by MAPKs (Wei and Seemann, 2009). Here, we used mass spectrometry and sequence searches to identify putative MAPK substrates in the Golgi proteins. To our surprise, we identified only eight such proteins (Figure 1), an amount which is lower than we expected. Therefore, we believe that our screen missed several such proteins, and the number of putative ERK1c substrates in the Golgi is greater than the number of proteins reported here. It should be noted, however, that this problem has no bearing on the main subject of this study which is the characterization of HOOK3 phosphorylation by ERK1c and AuroraA. Another point, that may be somewhat problematic, is the relatively low level of fragmentation derived by ERK1c overexpression (∼27%). This may cause incomplete assessments of the role of some of the examined proteins in Figure 1. However, this method was chosen to validate the specificity of the ERK1c effects, and as mentioned above, has no bearing on the main issue of the current manuscript.

## STAR★Methods

### Key resources table

**Table:** 

REAGENT or RESOURCE	SOURCE	IDENTIFIER
**Antibodies**		

gERK	Sigma-Aldrich	Cat # M5670
Tubulin	Sigma-Aldrich	Cat # T4026
GAPDH	Santa Cruz Biotechnology	Cat # SC-25778
Cyclin B1	Santa Cruz Biotechnology	Cat # 4138
GFP mouse monoclonal	Roche	Cat # 11814460001
GFP rabbit polyclonal	Abcam	Cat # ab290
GM130 (1)	Abcam	Cat # ab_52649
GM130 (2)	MBL	Cat # M179-3
CLASP2	Abcam	Cat # ab_95373
Aurora A	Abcam	Cat # ab_13824
phospho Ser/Thr-Pro	Abcam	Cat # ab_9344
phospho His H3.3 S31P	Abcam	Cat # ab 92628
pHis-H3 S28-AF488 conjugated antibody	BD Pharminigen	Cat # 558610
CCDC86	Bethyl Laboratories	Cat # A302-482A
VCP	Cell signaling technology	Cat # 2648
PAK4	Cell signaling technology	Cat #3242
HOOK3	Proteintech	Cat # 15457-1-AP
GRASP55	Proteintech	Cat # 10598-1-AP
ERK1c	(Aebersold et al., 2004)	Aebersold, D.M., Shaul, Y.D., Yung, Y., Yarom, N., Yao, Z., Hanoch, T. and Seger, R. (2004) Extracellular signal-regulated kinase 1c (ERK1c), a novel 42-kilodalton ERK, demonstrates unique modes of regulation, localization, and function. *Mol. Cell. Biol.***24**, 10000-10015.

**Critical commercial assays**		

Duolink PLA kit	Sigma	Cat # DUO92008
microtubule-associated protein spin-down kit (Cytoskeleton)	Cytoskeleton INC	Cat # BK029
RNeasy mini kit	Qiagen	Cat # 74106
The developing substrate NBT/BCIP	ADharmafect bcam	Cat # ab7468
Dharmafect	Thermo Fisher Scientific	Cat # T-2001-03

**Experimental models: cell lines**		

Human HAP1 Cells	Horizon Discovery	Cat # C631
Human HAP1 Cells HOOK3 KO	Horizon Discovery	Cat # 84376
HeLa cells	ATCC	Code # CCL-2

**Recombinant DNA**		

GFP-ERK1	Shaul and Seger (2006)	Shaul, Y.D. and Seger, R. (2006) ERK1c regulates Golgi fragmentation during mitosis. *J. Cell Biol.***172**, 885-897.
GFP-ERK1c	Shaul and Seger (2006)	Shaul, Y.D. and Seger, R. (2006) ERK1c regulates Golgi fragmentation during mitosis. *J. Cell Biol.***172**, 885-897.
GFP-HOOK3 WT	This article	This article
GST-HOOK3	This article	This article

**Other**		

PD184352	Selleckchem	Cat # CI-1040
[γ32P]-ATP	Perkin-Elmer	Cat # BLU502A250UC
Draq5	BioStatus	Cat # DR05500
DAPI	Termo Fisher	Cat # D1306

### Resource availability

#### Lead contact

Further information and requests for resources and reagents should be directed to and will be fulfilled by the lead contact, Rony Seger (rony.seger@weizmann.ac.il).

#### Materials availability

Reagents generated in this study are available by request.

#### Data and code availability

The published article includes all [datasets/code] generated or analyzed during this study.

### Experimental models and subject details

We used here HeLa cells that were cultured in Dulbecco’s modified Eagle’s medium (DMEM), supplemented with 10% fetal bovine serum (FBS), 2 mM L-glutamine and 1% Pen/Strep. Human HAP1 WT and HOOK3 KO cells were obtained from Horizon Discovery (USA) and were grown in Iscove's modified Dulbecco's medium (IMDM) supplemented with 10% fetal bovine serum (FBS), 2 mM L-glutamine and 1% Pen/Strep. All cells were maintained at 37°C in a humidified atmosphere of 95% air and 5% CO_2_. Cells were transfected with plasmids using polyethylenimine and with SiRNAs using Dharmafect. Cells were synchronized at the G1/S boundary by the double thymidine block. In short, cells were treated with 2.5 mM thymidine in DMSO, washed twice with PBS, grown for 8 h in regular medium, treated again with 2.5 mM thymidine for additional 16 h and then washed with PBS. This marks time 0, after which the cells were grown regularly. Cells were also synchronized at the M phase, using 100 ng/ml nocodazole for 16 h. No subjects were used.

### Method details

#### RNA extraction and RT-PCR

Total RNA was isolated from GFP-ERK1 or GFP-ERK1c transfected HeLa cells which were further treated with the indicated SiRNAs. RNA was isolated using RNeasy mini kit (Qiagen, MD, USA) according to the manufacture’s instruction and reverse transcribed into cDNA using Superscript III (Invitrogen, CA, USA). PCR was performed with the specific target genes’ primers and for actin as control using Taq high fidelity PCR enzyme mix (Thermo Fisher Sceintific MA, USA).

#### Immunoprecipitation (IP) and coIP

Cells after stimulation or other treatments were rinsed twice with ice-cold phosphate buffered saline (PBS) and once with Buffer A. The cells were then scraped into Buffer H (0.4 ml/plate), sonicated (50 W, 2 × 7 sec), and centrifuged (15,000 x g, 15 min). Supernatants were then incubated for 2 hr (4°C, under rotation) with A/G-agarose beads (Santa Cruz Biotechnology, Dallas, Texas) pre-linked with specific antibodies (1 hr, 23°C). For coIP, the bound A/G beads were washed three times with ice-cold coIP-washing buffer or with RIPA buffer, twice with 0.5 M LiCl_2_ and twice with Buffer A for IP. The IPed beads were then resuspended with sample buffer and boiled; the resolved proteins were analyzed by Western blotting with the indicated antibodies.

#### *In vitro* kinase assay

Immunoprecipitated ERKs or AuroraA attached to A/G beads were used as kinases by mixing them with GST-HOOK3 (5 μg/reaction). Buffer RM containing 100 μM γ[32P]ATP (4,000 cpm/pmol), was added to the reaction at a final volume of 30 μL and incubated for 20 min at 30°C under shaking. The reaction was terminated by adding 10 μL of 4X sample buffer, and the phosphorylated proteins were resolved on SDS-PAGE and subjected to autoradiography and Western blot analysis with the proper antibodies.

#### Immunofluorescence microscopy

Cells were fixed in 4% paraformaldehyde and in PBS (20 min, 23°C), and permeabilized with Triton X-100 (0.1% Triton X-100, 2% BSA in PBS, 5 min, 23°C), as previously described (Chuderland et al., 2008). The cells were then incubated with the primary antibodies (1 hr, 23°C), washed three times with PBS and incubated with secondary antibodies (1 hr, 23°C), and DAPI. Slides were visualized by Spinning Disk Confocal Microscopy (x60 magnification Ziess, Jena, Germany). Background correction and contrast adjustment were done with Photoshop (Adobe, CA, USA).

#### Proliferation assay

Cell proliferation was determined using a Methylene Blue assay. For this purpose, HAP1 WT and KO cells as well as SiRNAs treated HeLa cells were grown for 72 or 96 hr in 1% FCS-containing medium. Cells were fixed in 4% formaldehyde at room temperature for 2 hr, then washed once in 0.1 M sodium borate buffer, pH 8.5, and, thereafter, incubated with 1% Methylene Blue in 0.1 M sodium borate buffer, pH 8.5, for 10 min. Excess of stain was washed out with 0.1 M sodium borate buffer, pH 8.5, and the stain was extracted with 0.1 M HCl (0.4 mL/well) for 20 min at room temperature with shaking. Aliquots were transferred into a 96-well plate, and absorbance at 495 nm was determined using an ELISA reader.

#### Multispectral imaging flow cytometry analysis

Synchronized cells were methanol fixed, and stained, as described above using HOOK3, CLASP2 and GM130 Abs, and the DNA was stained using DAPI or Draq5. Cells were imaged using multispectral imaging flow cytometry (ImageStreamX flow-cytometer; Amnis Corp, Seattle, WA). For multispectral imaging flow cytometry, approximately 1-2x10^4^ cells were collected from each sample and data were analyzed using image analysis software (IDEAS 4.0; Amnis Corp). Images were compensated for fluorescent dye overlap by using single-stain controls. Cells were gated for single cells, using the area and aspect ratio features, and for focused cells, using the Gradient RMS feature, as previously described (George et al., 2006). Cells were gated for G2/M based on the DNA intensity. The G2/M population was further gated for prophase, prometaphase and mitotic (metaphase-telophase) populations using 2 parameters: the bright detail intensity of DNA staining (intensity of localized bright areas, subtracted for the local background), and the area of the 50% highest intensity pixels of the Draq5 staining. The different mitotic stages were further defined by the nuclear morphology, as previously described (Merrill, 1998). Colocalization was quantified, using the bright detail similarity feature (the log transformed Pearson's correlation coefficient of the localized bright spots in the two input images (George et al., 2006). Images of cells with similarity values above 1.5 were considered to display colocalization. To quantify Golgi fragmentation by IFC, two features were eventually chosen based on the GM130 staining: Minor Axis Intensity (the intensity weighted narrowest dimension of the ellipse of best fit) and Area (the number of microns squared within a mask). These were calculated on the Threshold_60 mask that includes the 60% highest intensity pixels of the GM130 staining. These were plotted as a bivariate plot and the different Golgi morphologies were gated according to visual inspection as described (Wortzel et al., 2017).

#### Proximity ligation assay

Protein-protein interactions were detected by using a Duolink PLA kit (Olink Bioscience, Uppsala, Sweden (Lowder et al., 2011), according to the manufacturer's protocol. Briefly, cells were grown, fixed, and permeabilized, as described for Immunofluorescence microscopy. The samples were then incubated with primary antibodies against two examined proteins (1 hr, 23°C), washed (0.01 M Tris-HCl, pH 7.4, 0.15 M NaCl, and 0.05% Tween 20), and then incubated with specific probes (1 hr, 37°C), followed by DAPI staining to visualize nuclei and then washed (0.2 M Tris-HCl pH 7.5, 0.15 M NaCl). Then the samples were incubated with anti pHis3 to detect mitotic cells. The signal was visualized as distinct fluorescent spots by using Spinning Disk Confocal Microscopy. The number of PLA events was counted automatically in ImageJ, by the ‘analyze particles’ feature. Each field was counted twice: first, for the number of nuclei in the field, and second, for the number of PLA events. Then the average number of events was calculated per treatment. More than 100 cells were counted per treatment. Background correction, contrast adjustment, and the quantification of the fluorescence signal were performed using Photoshop and ImageJ software.

#### Golgi extraction on non-continues sucrose gradient

Cells from three 15 cm^2^ confluent plates were harvested in buffer H. Cells were centrifuged for 4 min, 1400 rpm, 4°C, and resuspended with 750μL buffer H. Homogenization of the cells was performed by 50 strokes with manual pestles, and 20 strokes of a 1 mL syringe, and then centrifuged for 6 min, 4000 rpm, 4°C. Noncontinuous sucrose gradient was prepared by loading 800μL of 0.5, 0.8, 1, 1.2 and 1.5M sucrose solutions in buffer H. The supernatant (800μL) was loaded on top of the gradient and the sample was centrifuged in an Ultra-centrifuge for 2 hr, 4°C, 100,000 x g (35,000 RPM SW-T41i rotor). Finally, 12 fractions of 400μL were collected carefully from the top.

#### MTs pull-down assay

Microtubule spin-down assays were performed using the microtubule-associated protein spin-down kit (Cytoskeleton) according to the manufacturer's instructions. Briefly, purified tubulin was assembled into microtubules for 20 min at 35°C in the presence of GTP and stabilized with Taxol. Assembled microtubules (10 μg) were incubated with 30–60 μg of cytosolic protein extract in total volume of 50 μL for 20 min at RT. Microtubules and associated proteins were pelleted at 100,000 x g through a 40% glycerol cushion buffer. Supernatant and pellet were dissolved in sample buffer.

#### DNA constructs and mutations

GFP-ERK1 and GFP-ERK1c (Shaul and Seger, 2006), GFP-HOOK3 WT and mutants were cloned in pEGFP-C1 (Clontech, Mountain View, CA). Point mutations were performed by site-directed mutagenesis kit (Qiagen). GST-HOOK3 WT and mutants were cloned in pGEX-4T1 vector (GE Healthcare, Buckinghamshire, UK). The GST protein was purified according to the manufacturer's instructions and eluted from the glutathione beads using 10 mM of reduced glutathione.

#### Buffers

Buffer A: 50 mM β-glycerophosphate (pH 7.3), 1.5 mM EGTA, 1 mM EDTA, 1mM dithiothreitol, and 0.1 mM sodium vanadate. Buffer H: 50 mM β-glycerophosphate (pH 7.3), 1.5 mM EGTA, 1 mM EDTA, 1mM dithiothreitol, and 0.1 mM sodium vanadate, 10 μg/mL aprotinin, 10 μg/mL leupeptin, 2 μg/mL pepstatin A. RIPA buffer: 137 mM NaCl, 20 mM Tris pH 7.4, 10% (v/v) glycerol, 1% Triton X-100, 0.5% (v/v) deoxycholate, 0.1% (w/v) SDS, 2 mM EDTA, 1 mM PMSF, and 20 mM leupeptin. RM solution: 10 mM MgCl2, 1.5 mM DTT, 25 mM β-glycerophosphate pH 7.3, 0.05 mM sodium vanadate, 1.25 mM EGTA, 10 μM calmidazolium, and 0.83 mg/mL bovine serum albumin (BSA).

#### Statistical analysis

Data are expressed as mean +S.E. Statistical evaluation was carried out using functional analysis and Student's T test (two-tailed) to test for differences between the control and experimental results. Values of ∗∗p < 0.01 or ∗p < 0.05 were considered significant.
