# Supplementary material for: Mitotic HOOK3 phosphorylation by ERK1c drives microtubule-dependent Golgi destabilization and fragmentation
Source: iScience. 2021 May 31;24(6):102670. doi: 10.1016/j.isci.2021.102670 (PMC8215223; doi:10.1016/j.isci.2021.102670)
Supplement: Document S1. Figures S1–S7 [file mmc1.pdf]

iScience, Volume *24*

Supplemental information

Mitotic HOOK3 phosphorylation by ERK1c drives microtubule-dependent Golgi destabilization and fragmentation

Inbal Wortzel, Galia Maik-Rachline, Suresh Singh Yadav, Tamar Hanoch, and Rony Seger

Supplemental figures

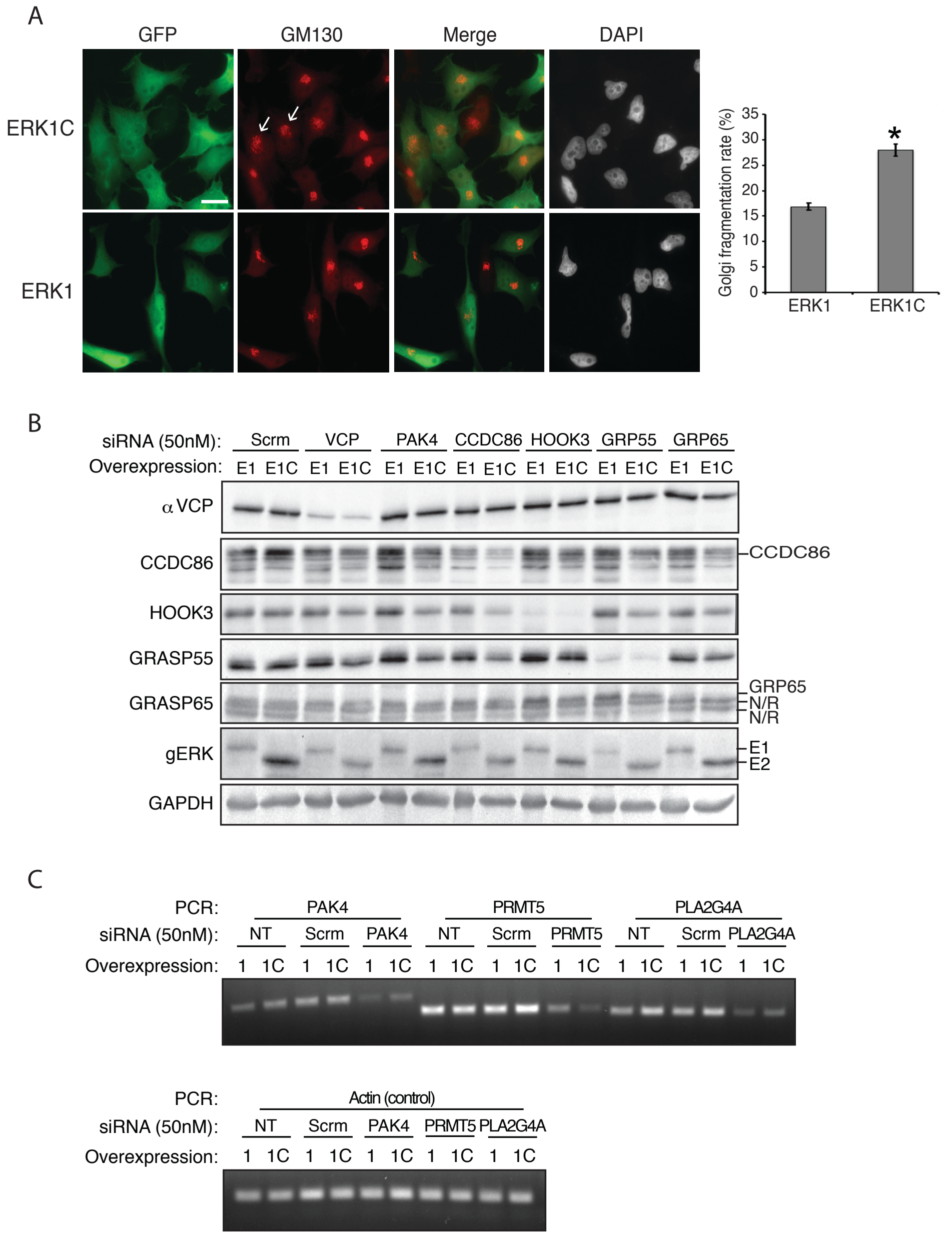

Figure S1. GFP-ERK1c induces Golgi fragmentation. Related to Fig. 1. (A) HeLa cells were transfected with GFP-ERK1c or GFP-ERK1 (green). The cells were fixed and stained for GM130 (red), and DAPI (white). Arrows indicate cells with fragmented Golgi. Scale bar, 10μm. (left panel). Golgi fragmentation was calculated as a percent of cells with fragmented Golgi, out of the overexpressed cells p<0.001. (Right panel). The experiment was reproduced more than 3 time. **(B)** SiRNA effect on the expression of ERK1c candidate substrates. HeLa cells were transfected with GFP-ERK1 or GFP-ERK1c for 24 hrs and then with 50 nM siRNA against the indicated proteins for additional 48 hrs. Total cell lysates were blotted against indicated proteins and GAPDH as loading control. GRP = GRASP, N/R = not relevant, E1 - ERK1, E1C – ERK1c. **(C)** SiRNA effect on the mRNA of additional ERK1c candidate substrates. For candidate substrates with no proper antibodies we established the effect of the knockdown in the mRNA level. For this purpose, HeLa cells were treated as described in panel B and subjected to RNA extraction followed by RT-PCR for the indicated genes (upper panel) or for actin as control (lower panel).

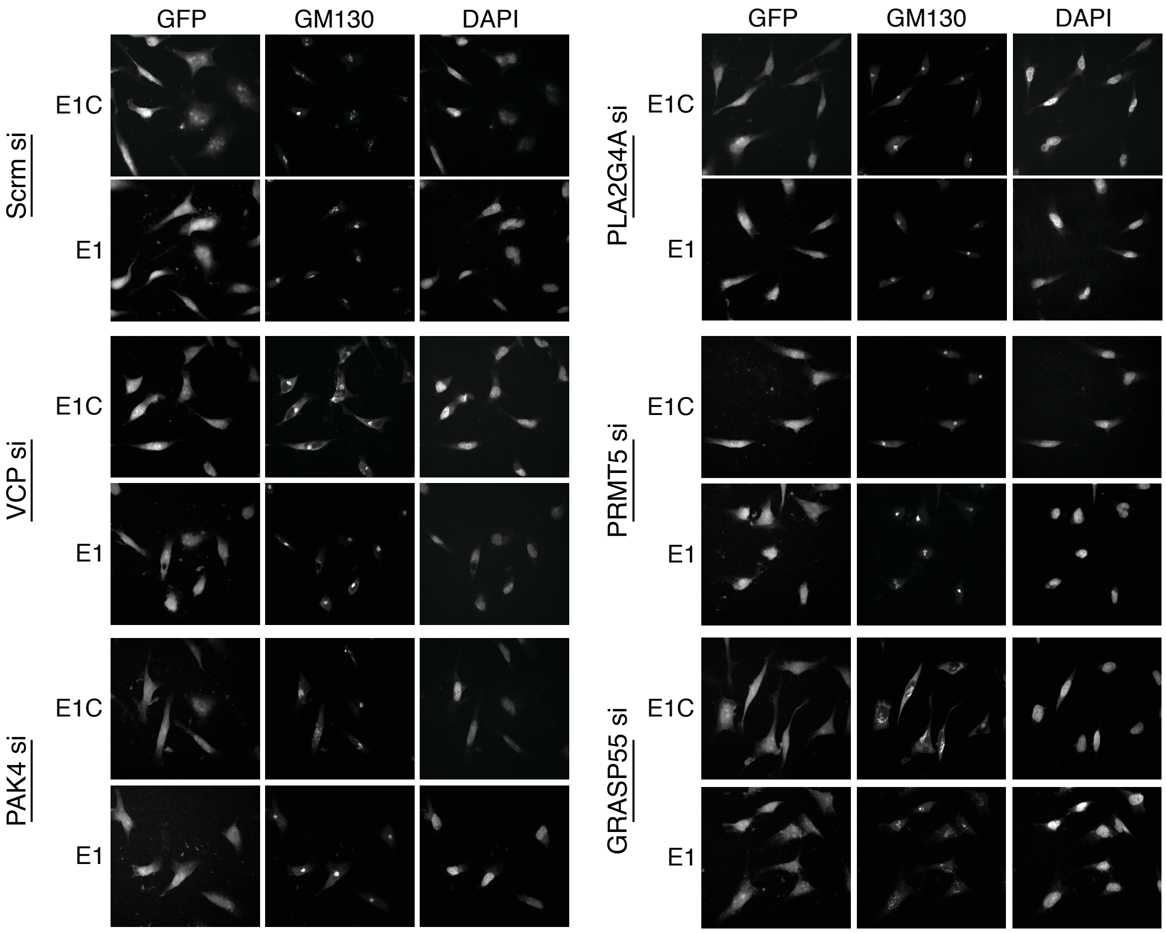

Figure S2. Effect of knockdown of the putative ERK1c substrates on ERK1c-induced Golgi fragmentation. Related to Fig. 1. HeLa cells were treated as above, fixed and stained with GM130 antibody. Images from the indicated SiRNA on HeLa cells, which were transfected with GFP-ERK1c (E1C) or GFP-ERK1 (E1) are shown. The results were reproduced 3 times.

A

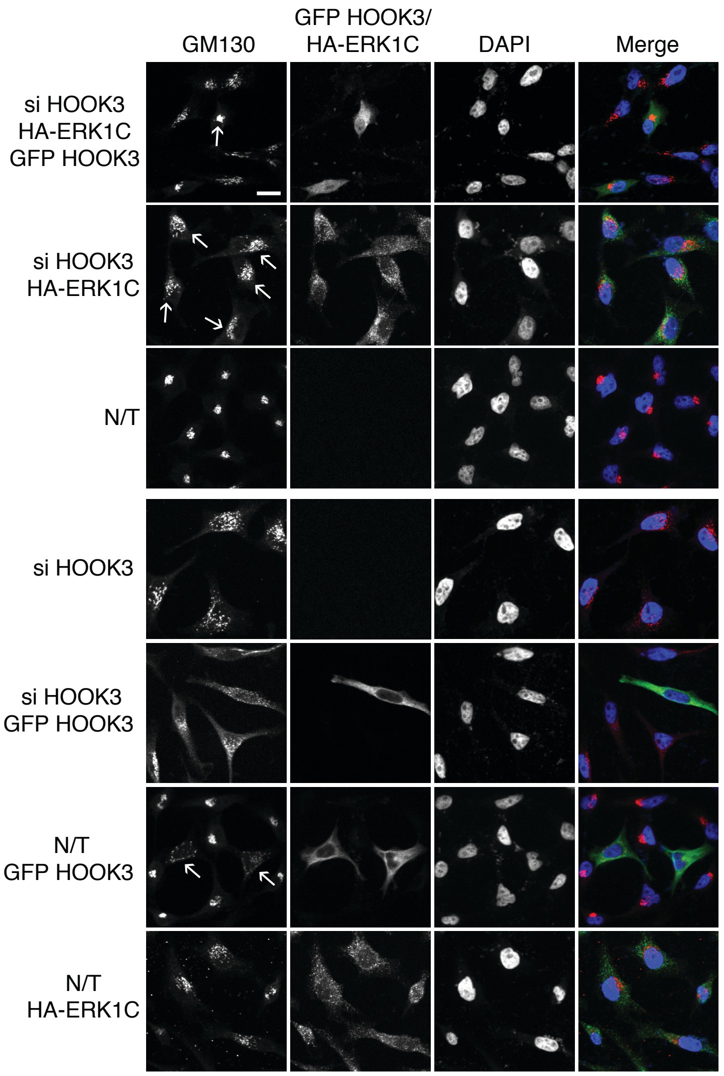

B

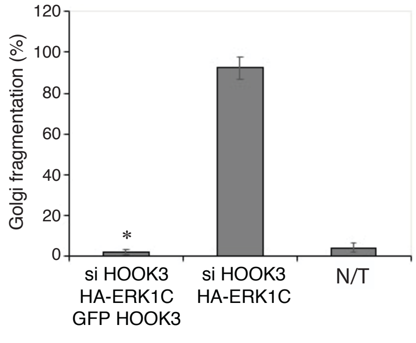

Figure S3. Overexpression of HOOK3 in the presence of ERK1c rescues its SiRNA effect. Related to Fig. 1. Representative images **(A)** and quantification **(B)** of fluorescence microscopy of GM130 (red), GFP-HOOK3 or HA-ERK1C (green), and DAPI (blue) staining in HeLa cells. HeLa cells were left non-treated (NT) or treated with HOOK3 SiRNA (Si HOOK3) for 24 hrs followed by transfection with GFP-HOOK3 or HA-ERK1c or co-transfection with both constructs for additional 48 hrs. Then cells were fixed and stained with the indicated antibodies. Merge images were taken using 3 channels following conformation of the existence of co-transfection. Scale bar, 10μm. N/T – non-treated. The bar-graph represents the averages and standard errors of percent of cells with fragmented Golgi out of the HA or GFP positive transfected cells. *$p<0.05$. The results were reproduced 3 times.

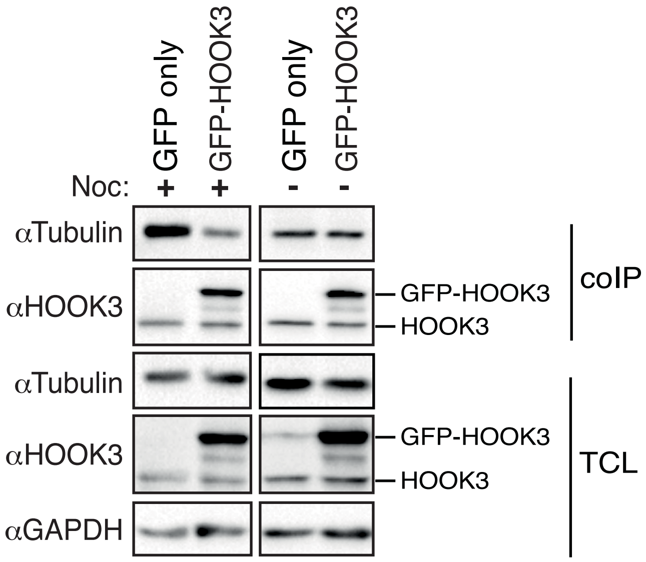

Figure S4. Overexpression of phosphorylated HOOK3 affects its microtubules interaction. Related to Fig. 5 and 6. Representative blots of coIP of tubulin with HOOK3 in HeLa cells transfected with GFP only or GFP-WT-HOOK3 (GFP HOOK3). Cells were treated with 100 ng/ml nocodazole for 16 hrs (+) or left non-treated (-). coIP – coimmunoprecipitation, TCL – total cell lysate.

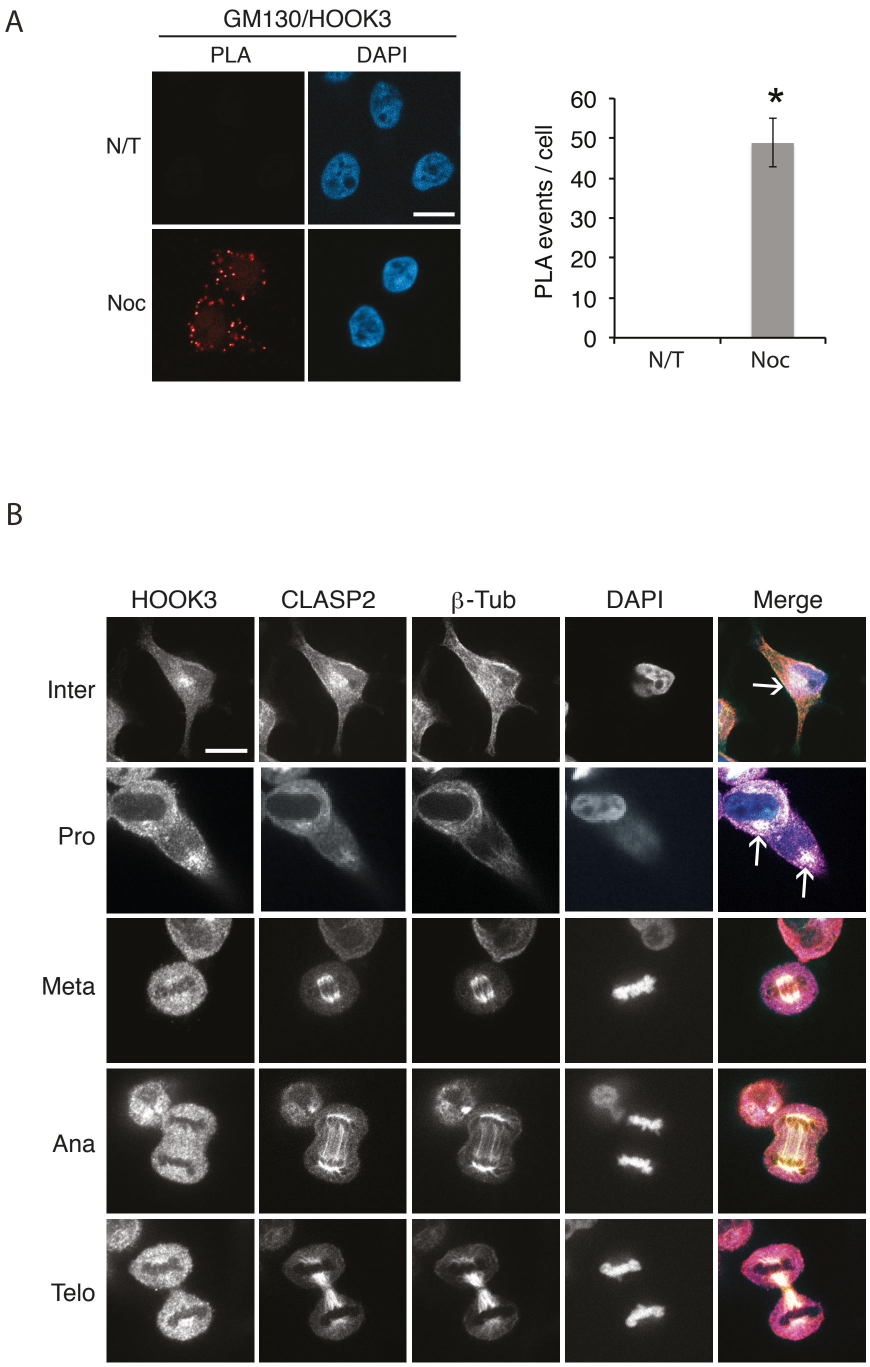

Figure S5. HOOK3 interacts with GM130 during mitosis. Related to Fig. 6. (A) Representative images and quantification of PLA analysis using anti GM130 and HOOK3 antibodies of HeLa cells which were treated with 100 ng/ml nocodazole for 16 hrs (Noc) or left non-treated (Con). The quantification was done as described under Materials and Methods. Scale bar, 10µm. **(B)** Representative images of fluorescence microscopy of HOOK3 (pink in the merge), CLASP2 (green), tubulin (β-Tub, red) and DAPI (blue) staining in synchronized HeLa cells. Cells were fixed at G1/S border (Inter) and at the peak of mitosis. Cells from prometaphase (Pro), metaphase (Meta), anaphase (Ana) and telophase (Telo) were selected by their DNA structure. All experiments were reproduced 3 times. Scale bar, 10µm. Arrows indicate staining's colocalization.

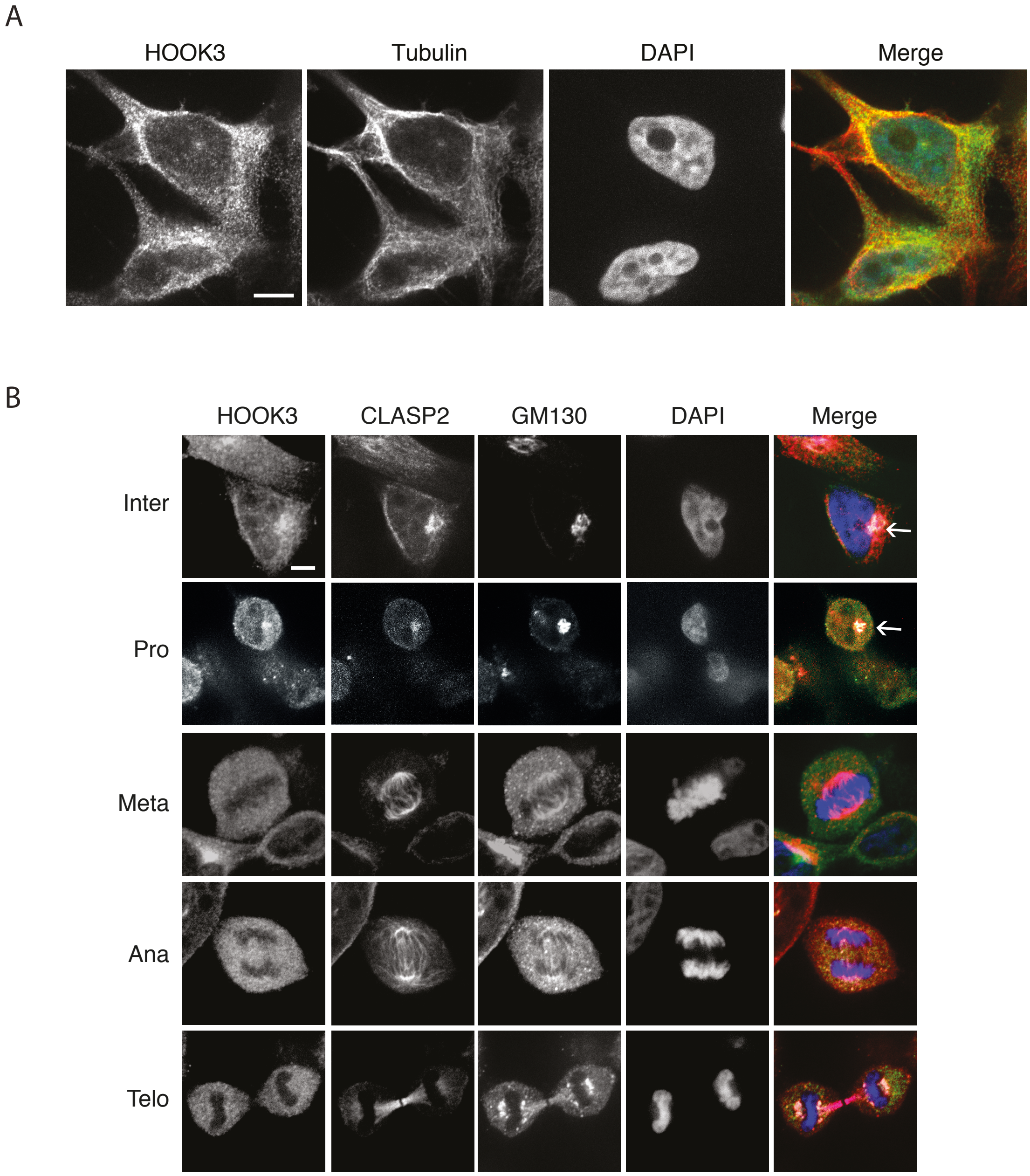

Figure S6. HOOK3 interacts with microtubules. Related to Fig. 6. (A) Representative images of fluorescence microscopy of HOOK3 (green), Tubulin (red), and DAPI (blue) staining in HeLa cells. Colocalization of HOOK and tubulin appeared yellow in the merge panel. Scale bar, 10µm. The experiments were reproduced twice. **(B)** Representative images of fluorescence microscopy of HOOK3 (pink in merge), CLASP2 (green), GM130 (red) and DAPI (blue) staining in HeLa cells. Cells were fixed at G1/S border (Inter) and at the peak of mitosis. Cells from prometaphase (Pro), metaphase (Meta), anaphase (Ana) and telophase (Telo) were selected by their DNA structure. Scale bar, 10µm. Arrow indicate staining's colocalization.

A

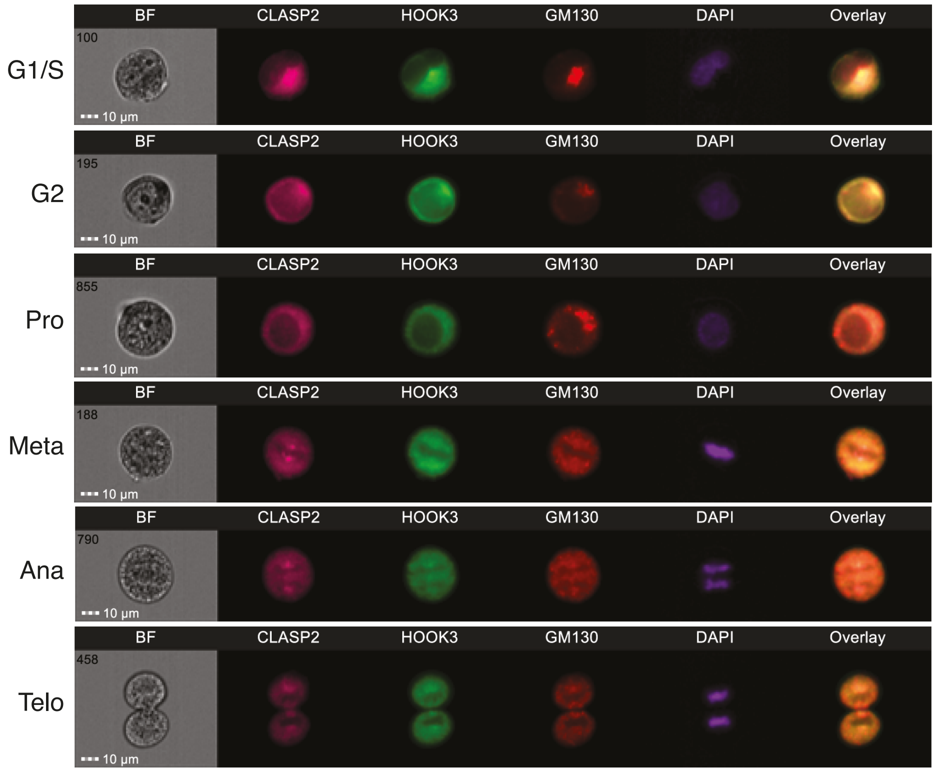

B

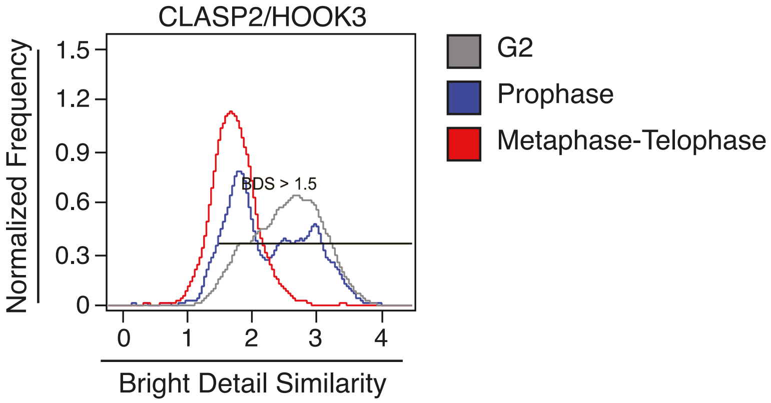

Figure S7. HOOK3 colocalizes with CLASP2 in cycling cells. Related to fig. 6. (A, B) Multispectral imaging flow-cytometry (Imagestream) images (A) and quantification (B) of HeLa cells that were stained with HOOK3 (green), CLASP2 (pink), GM130 (red) and DAPI (blue) in cycling HeLa cells (G1/S, G2) and in different stages of mitosis. Cells from G1/S, G2, prometaphase (Pro), metaphase (Meta), anaphase (Ana) and telophase (Telo) were selected by their DNA structure according to their DAPI staining. The results were reproduced 3 times.
